# Supplementary material for: Therapeutic targeting SPI1 in combination with erastin promotes ferroptosis in ccRCC
Source: Commun Biol. 2025 Dec 10;8:1772. doi: 10.1038/s42003-025-08900-4 (PMC12708875; doi:10.1038/s42003-025-08900-4)

Supplementary figure 1

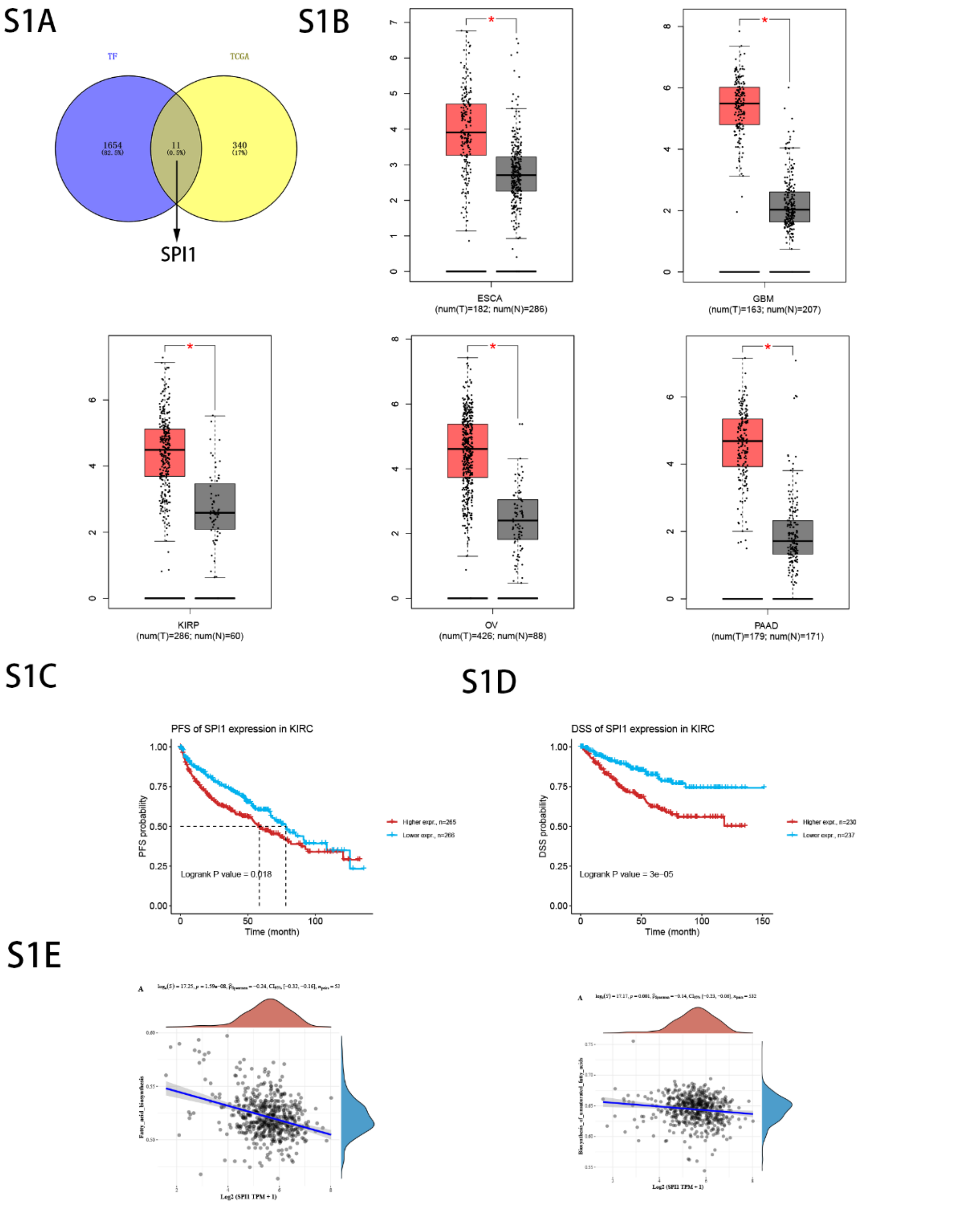

(S1A) Cross-Venn diagram of genes in the TCGA-KIRC differentially highly expressed gene set (Log2FC ≥ 2, q < 0.01) and the ENCODE database gene set. (S1B) SPI1 mRNA expression in a wide range of tumors from TCGA database, including ECSA, GBM, KIRP, OV and PAAD. (S1C-S1D) The SPI1 Kaplan–Meier survival curves for PFS and DSS are presented from TCGA-KIRC. (S1E) Correlation analysis of SPI1 mRNA expression and unsaturated fatty acid. *: P<0.05, **: P<0.01, ***: P<0.001

Supplementary figure 2

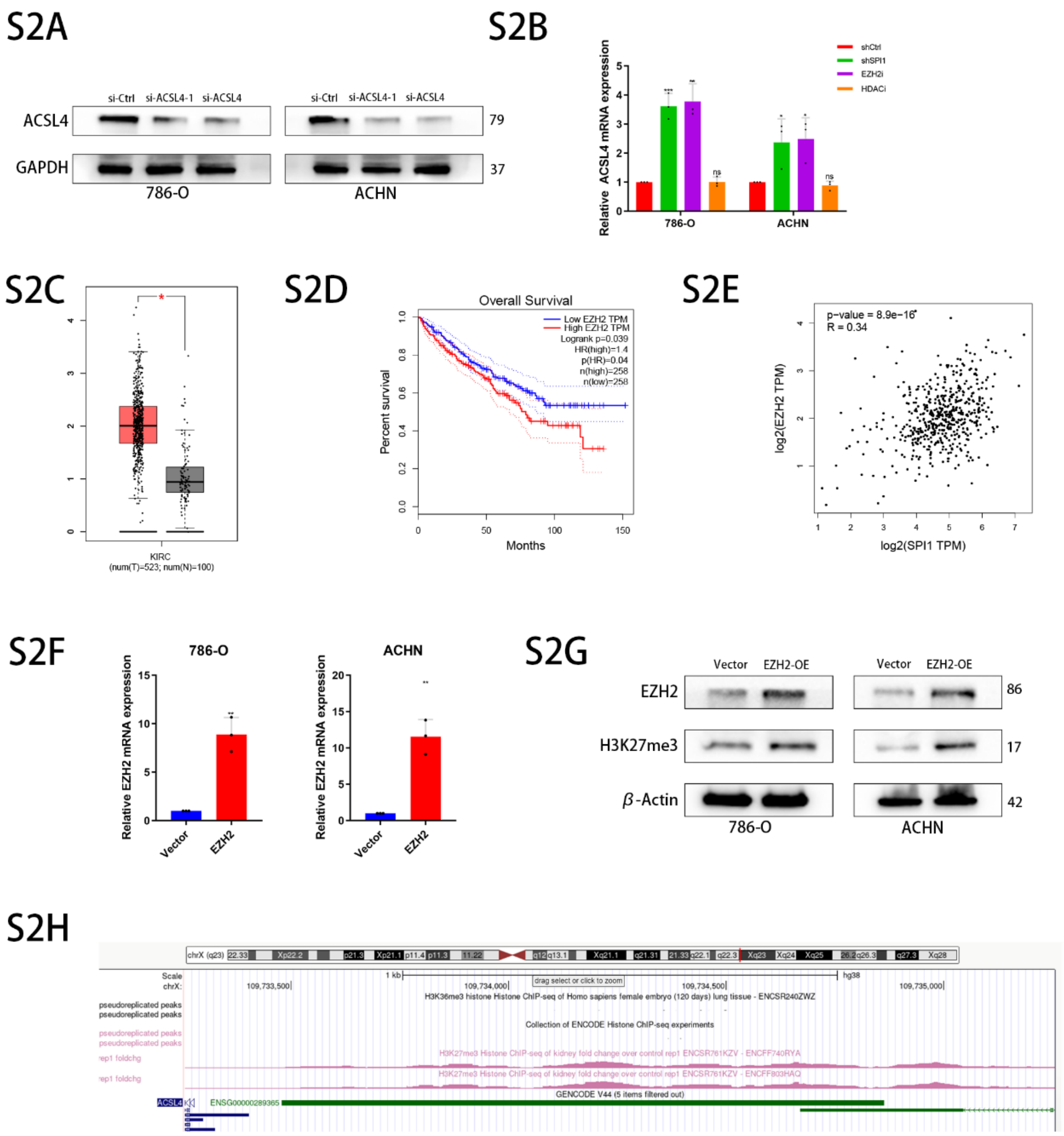

(S2A) Western blot examined the efficiency of ACSL4 knockdown in 786-O and ACHN. (S2B) qRT-PCR analysis of ACSL4 mRNA in 786-O and ACHN cells treated with shCtrl, shSPI1, UNC1999 (5 μM) and Entinostat (300 nM). (S2C) The mRNA expression of EZH2 in normal renal tissues and renal cancer tissues from TCGA-KIRC database. (S2D) Overall survival (OS) Kaplan–Meier curve for EZH2 based on TCGA-KIRC. (S2E) Correlation of SPI1 mRNA and EZH2 mRNA from TCGA database. (S2F, S2G) qRT-PCR and western blot examined the efficiency of EZH2 overexpressing in 786-O and ACHN. (S2H) H3K27me3 binding in the ACSL4 promoter region H3K27me3 related ChIP-seq sequencing from UCSC.

Supplementary figure 3

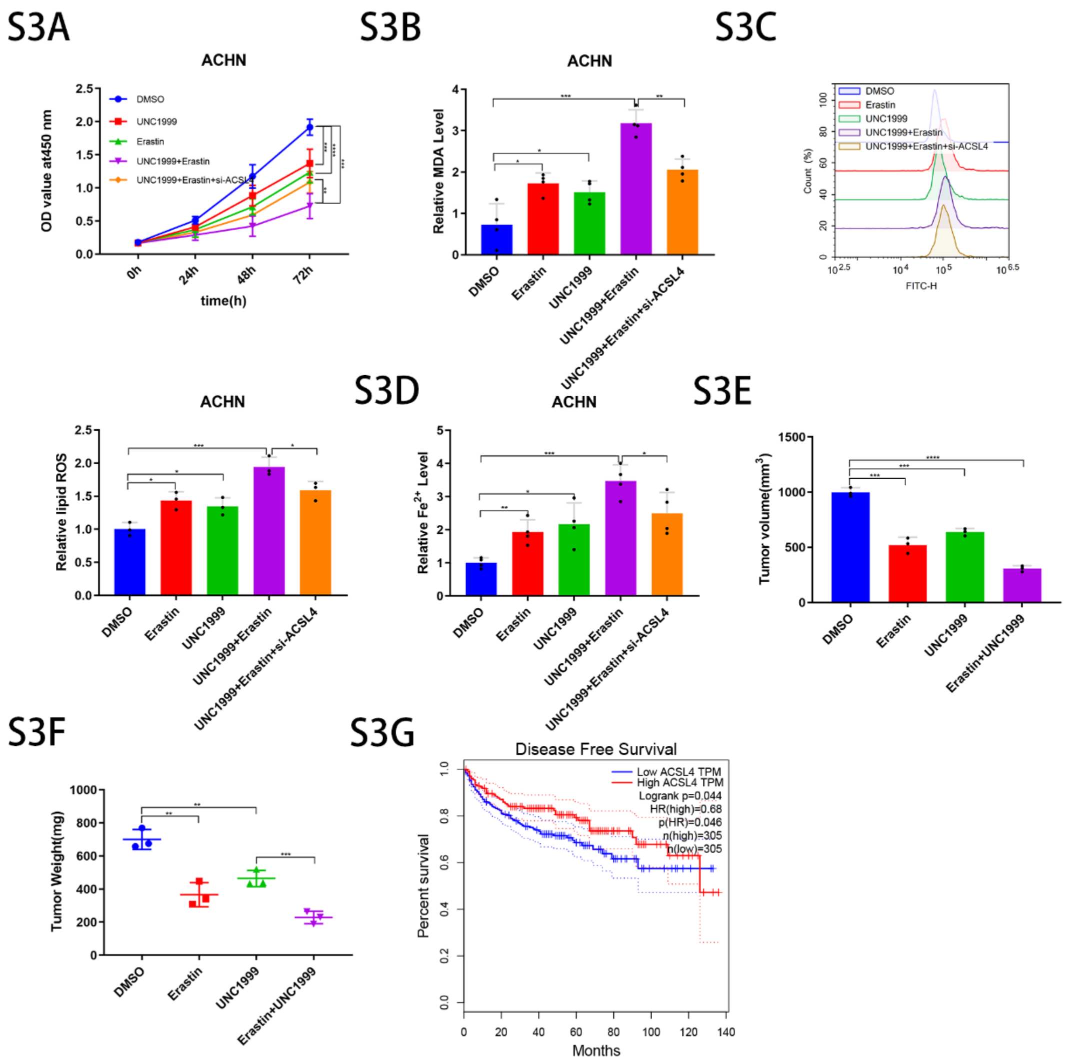

(S3A) CCK-8 assays were used to analyse the effect in the DMSO, UNC1999, Erastin, UNC1999+Erastin as well as UNC1999+Erastin+si-ACSL4 groups on cell viability in ACHN. (S3B) MDA levels were measured separately in the DMSO, UNC1999, Erastin, UNC1999+Erastin as well as UNC1999+Erastin+si-ACSL4 groups in ACHN. (S3C) Flow cytometry was used to detect lipid peroxidation level in the DMSO, UNC1999, Erastin, UNC1999+Erastin as well as UNC1999+Erastin+si-ACSL4 groups in ACHN. (S3D) Fe^{2+} levels were measured separately in the DMSO, UNC1999, Erastin, UNC1999+Erastin as well as UNC1999+Erastin+si-ACSL4 groups in ACHN. (S3E, S3F) The final tumor volume and weight were measured at 26-day (S3G) The ACSL4 Kaplan–Meier survival curves for DFS are presented from TCGA-KIRC. *: P<0.05, **: P<0.01, ***: P<0.001

Supplementary figure 4

Result-1（1L)

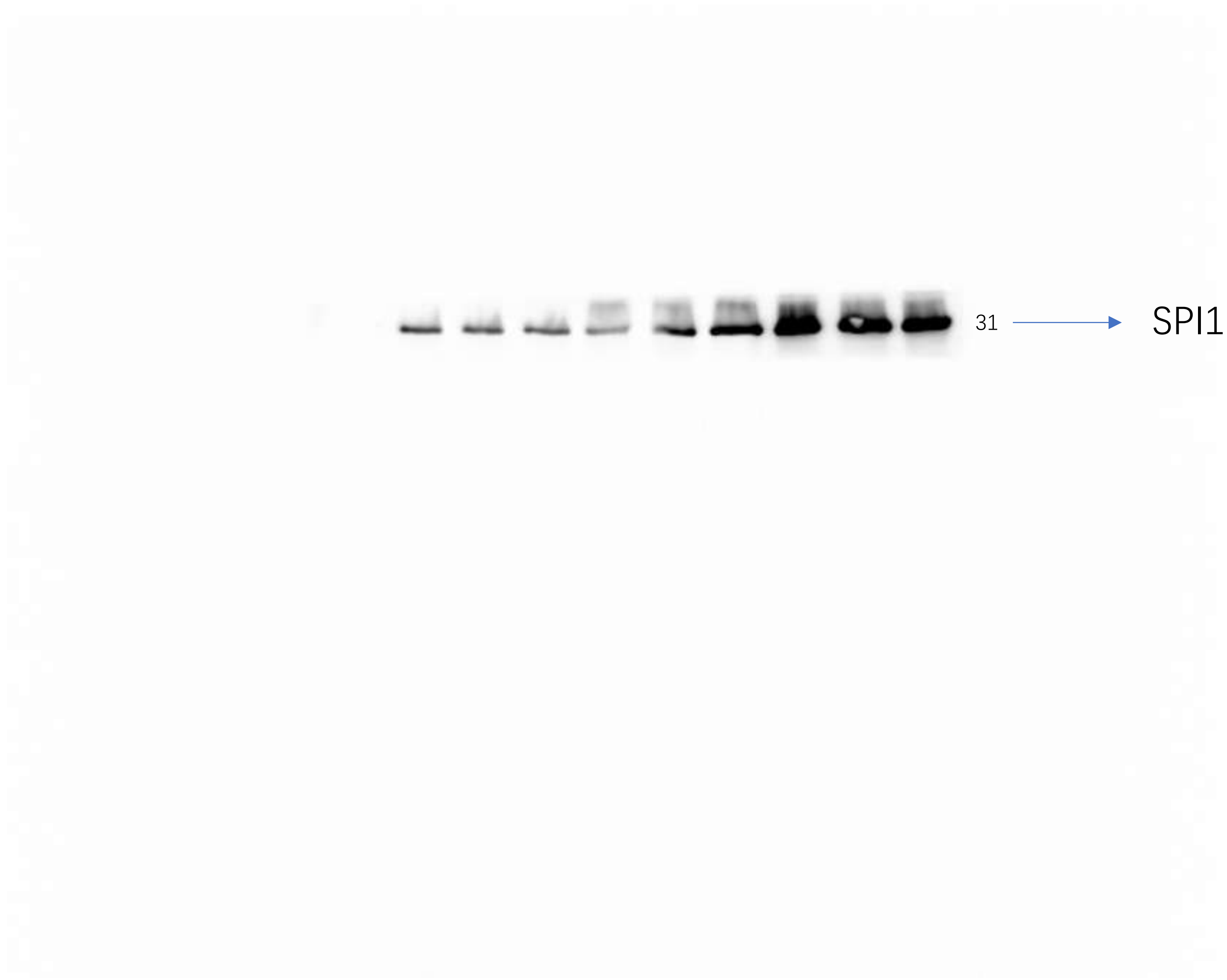

Result-1（1L)

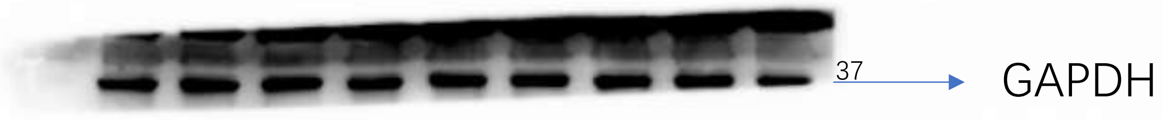

Result-1（1E）

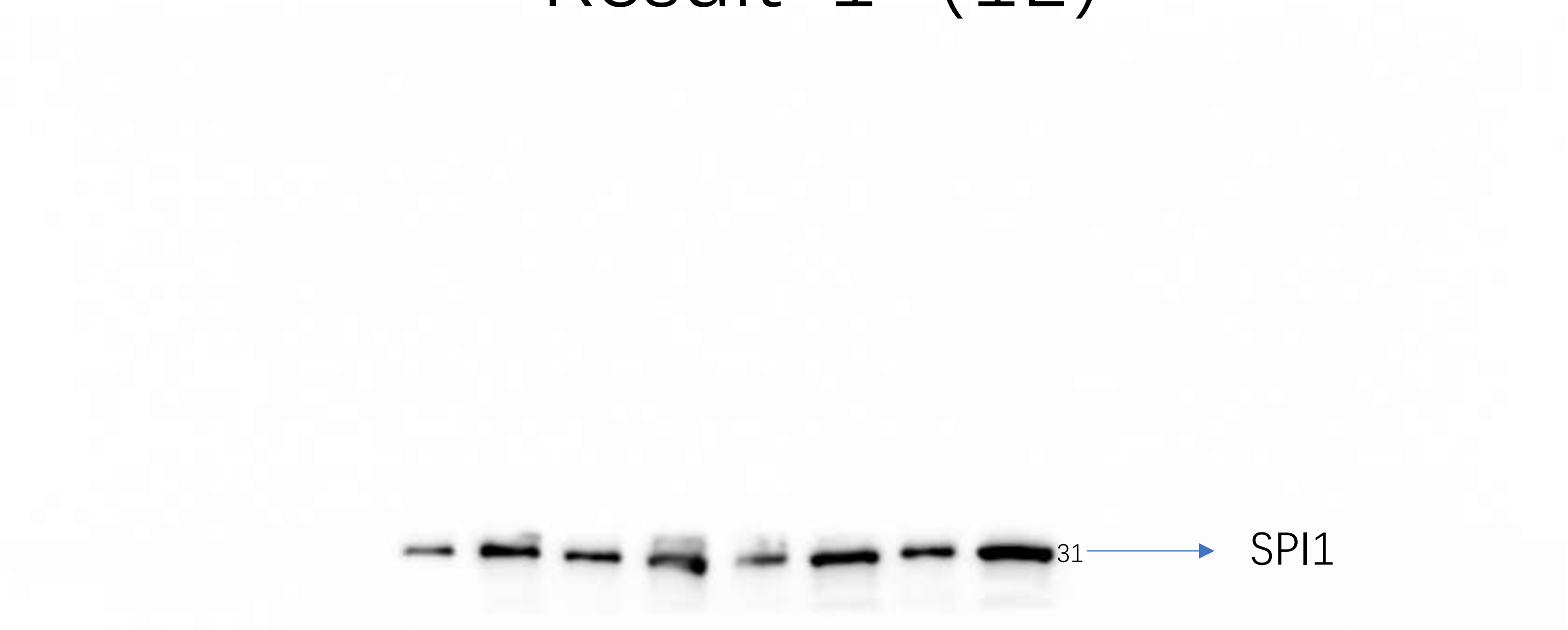

Result-1（1E)

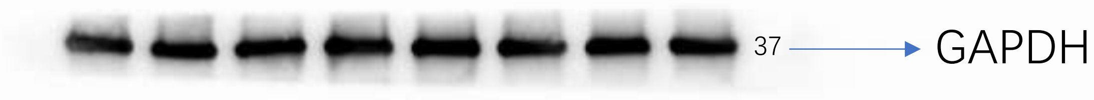

Result-1（1G）

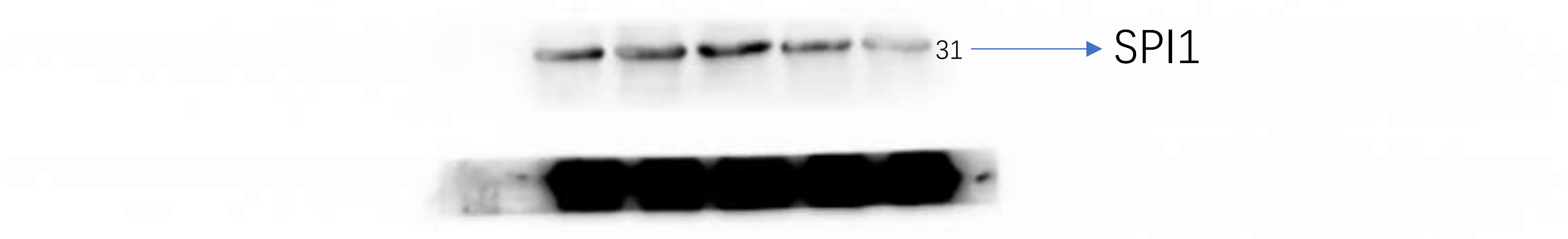

Result-1（1G）

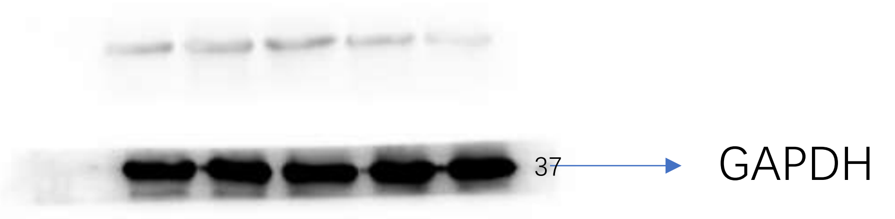

Result-2（2C）

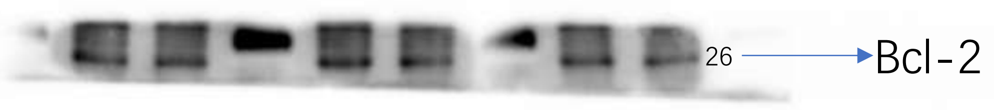

Result-2（2C)

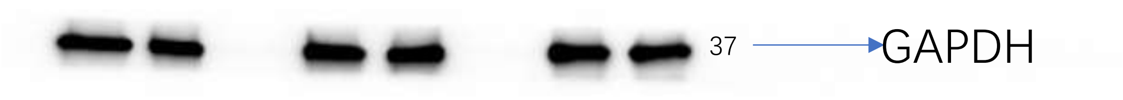

Result-2（2C）

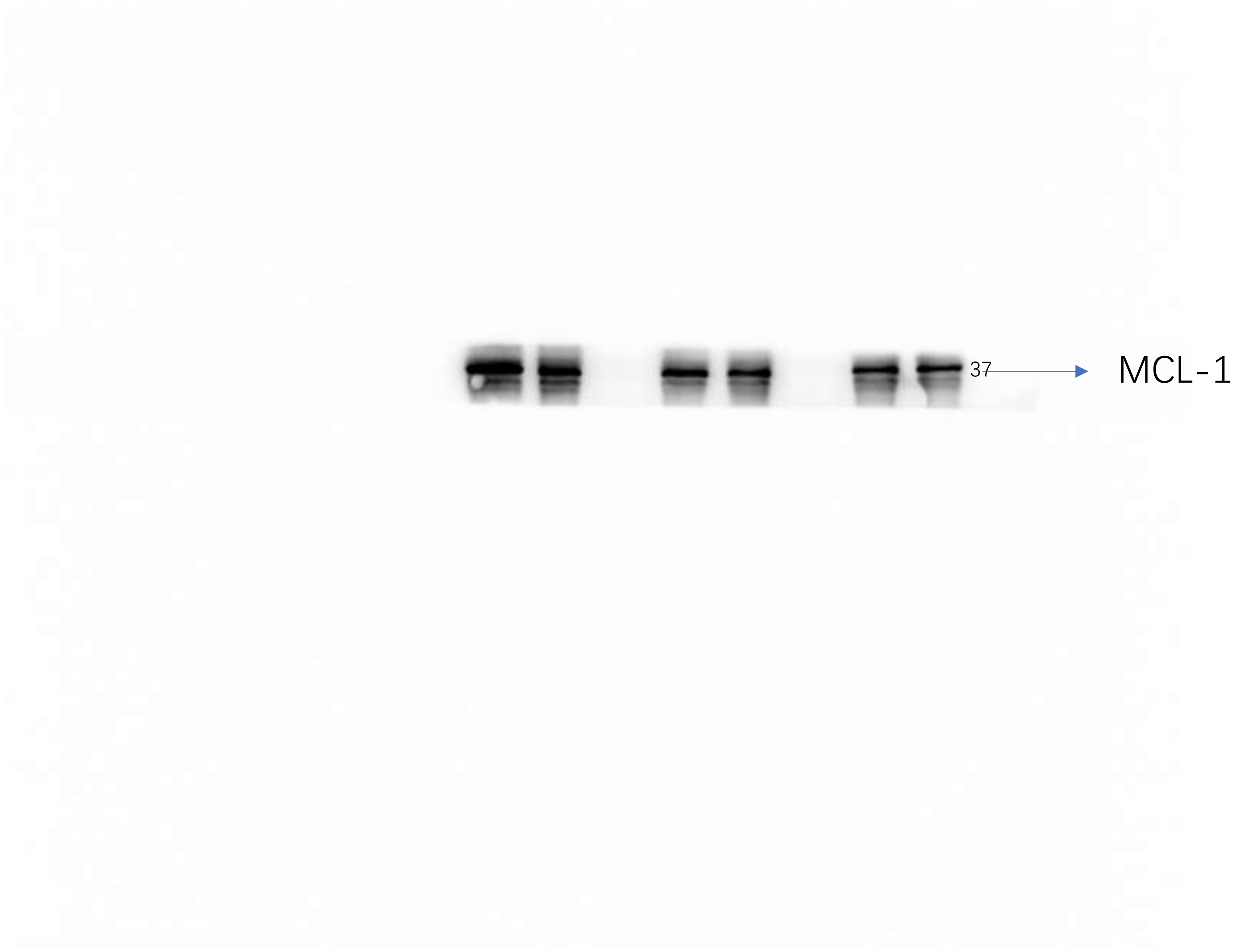

Result-2（2C）

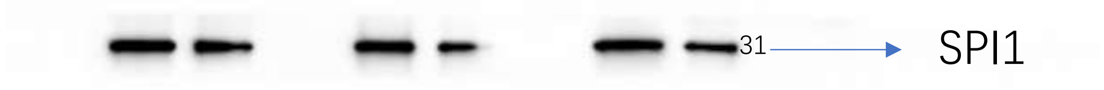

Result-2（2C)

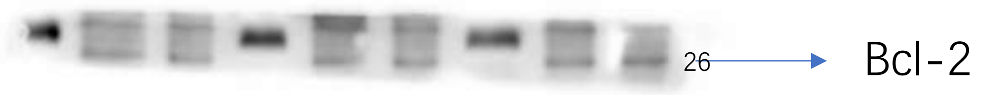

Result-2（2C)

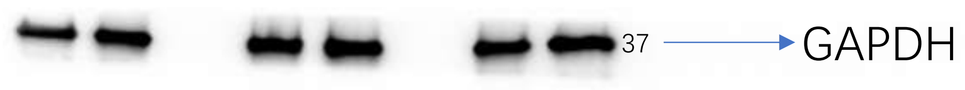

Result-2（2C）

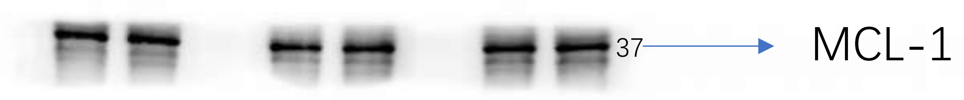

Result-2（2C）

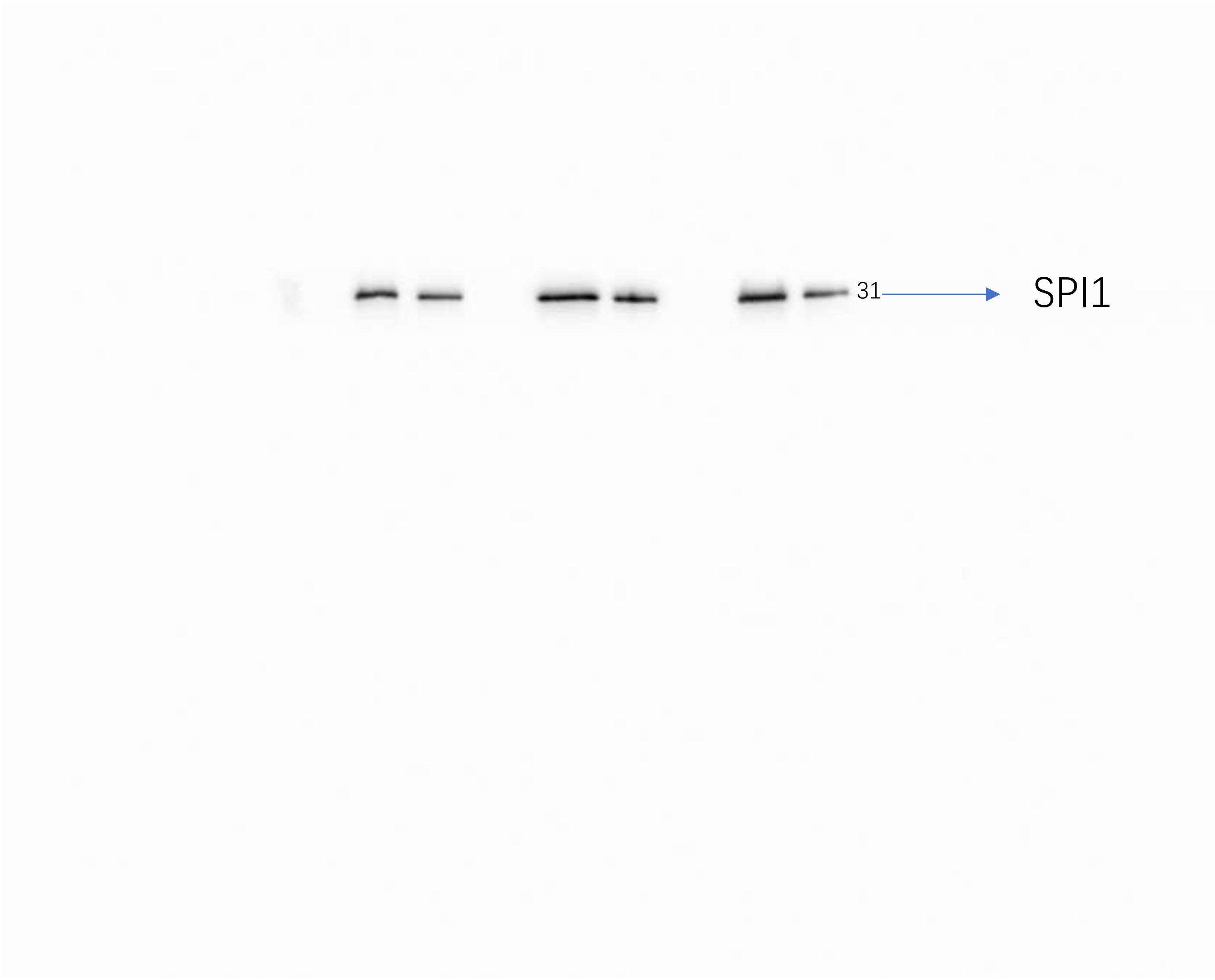

Result-2（2B）

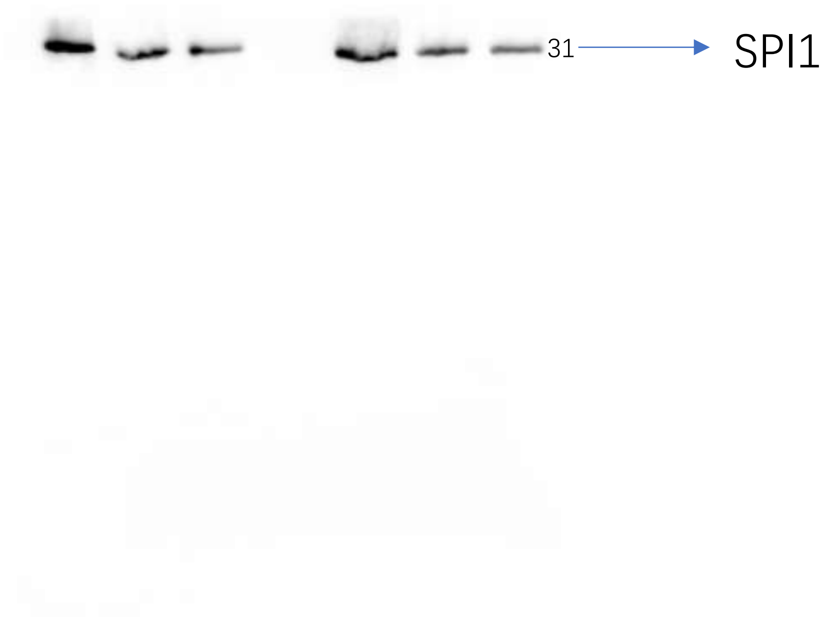

Result-2（2B）

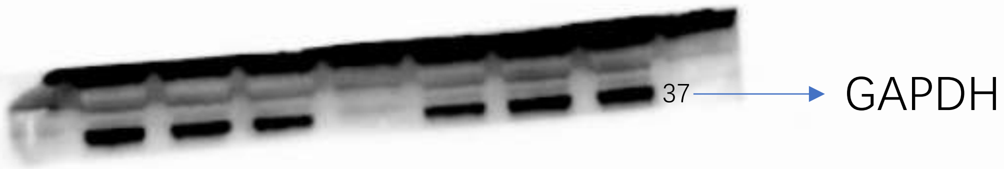

Result-3（3A）

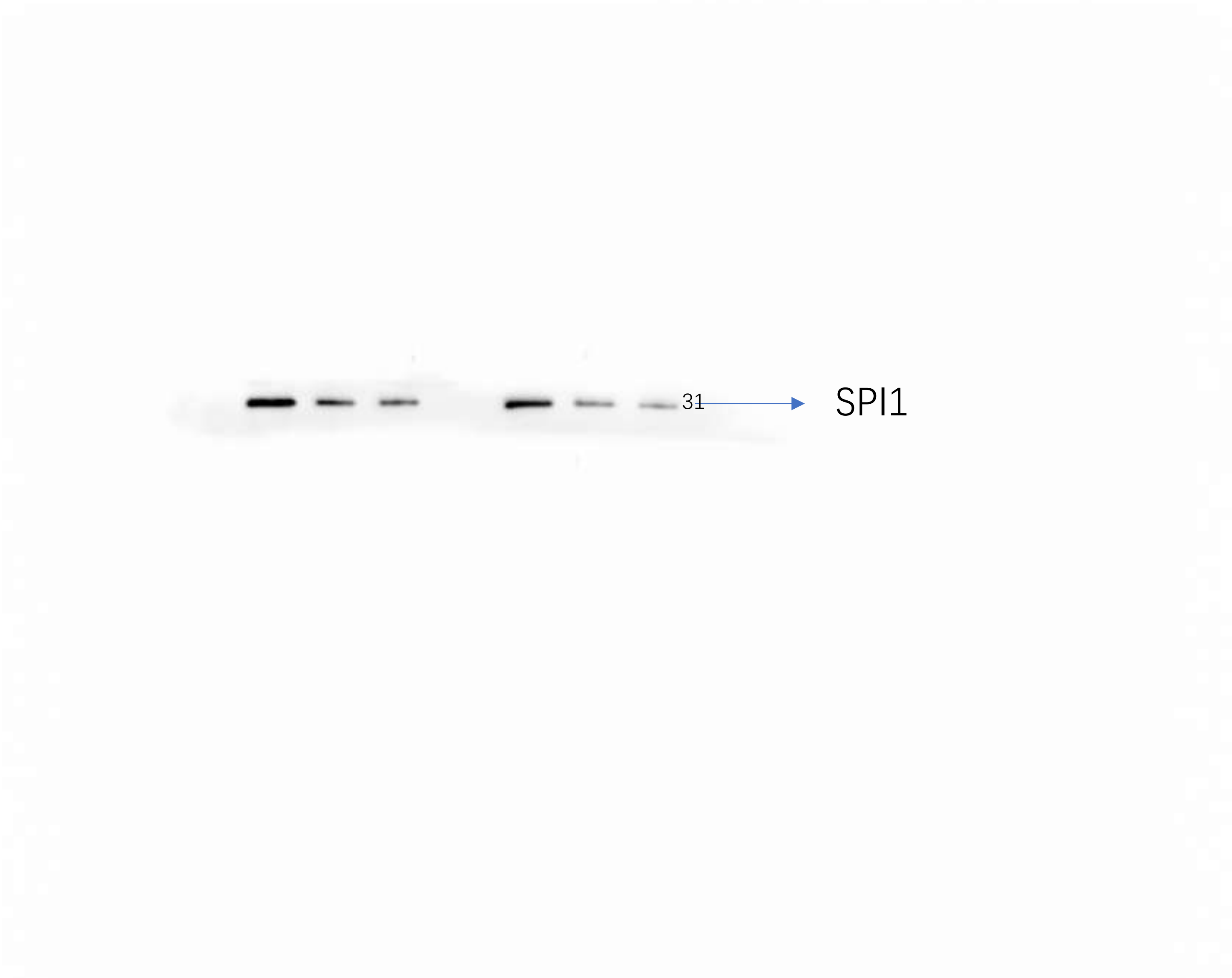

Result-3（3A）

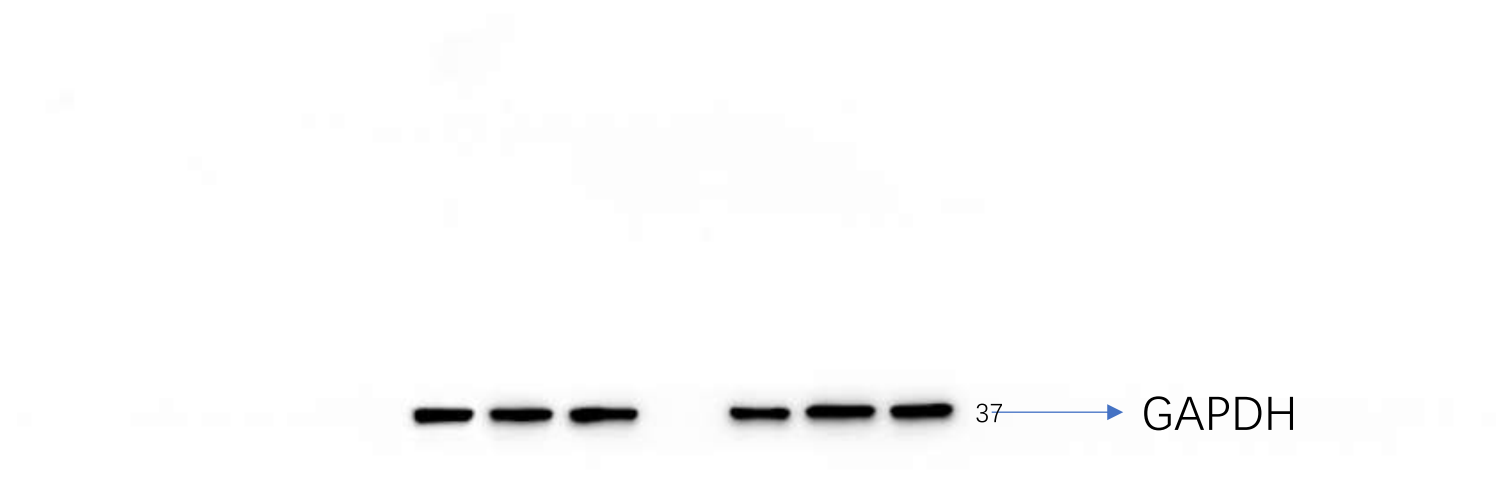

Result-3（3C）

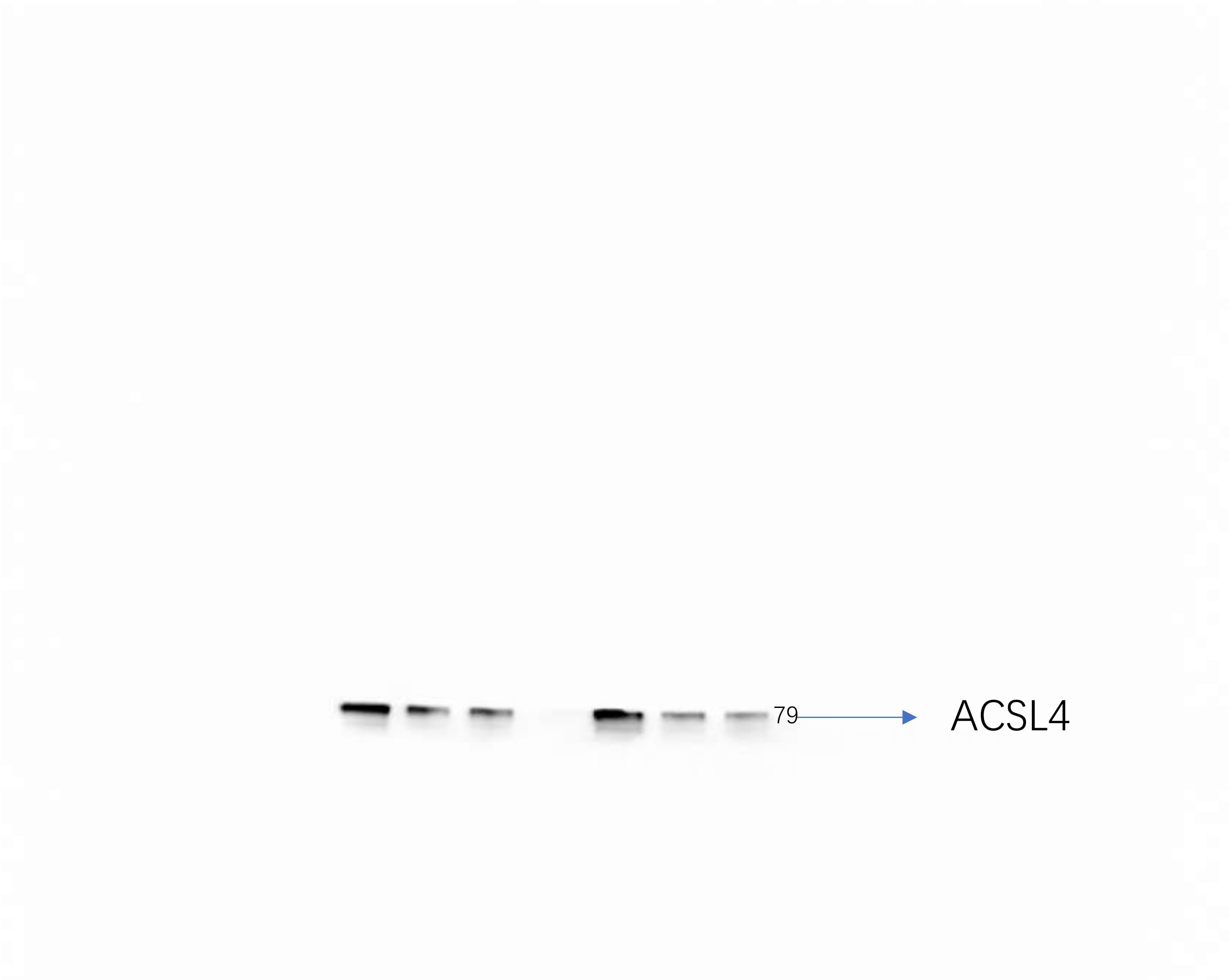

Result-3（3C）

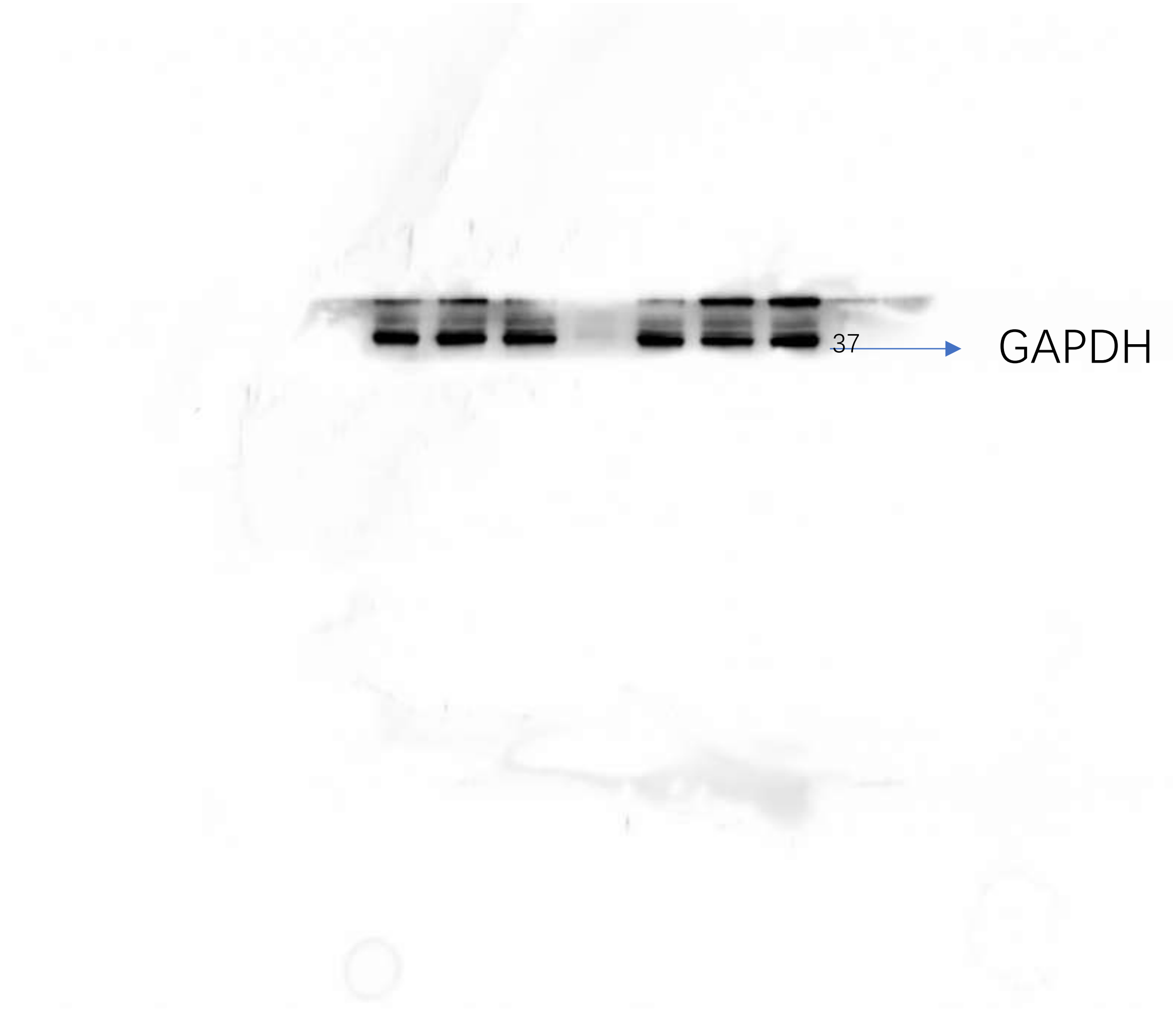

Result-4（4F）

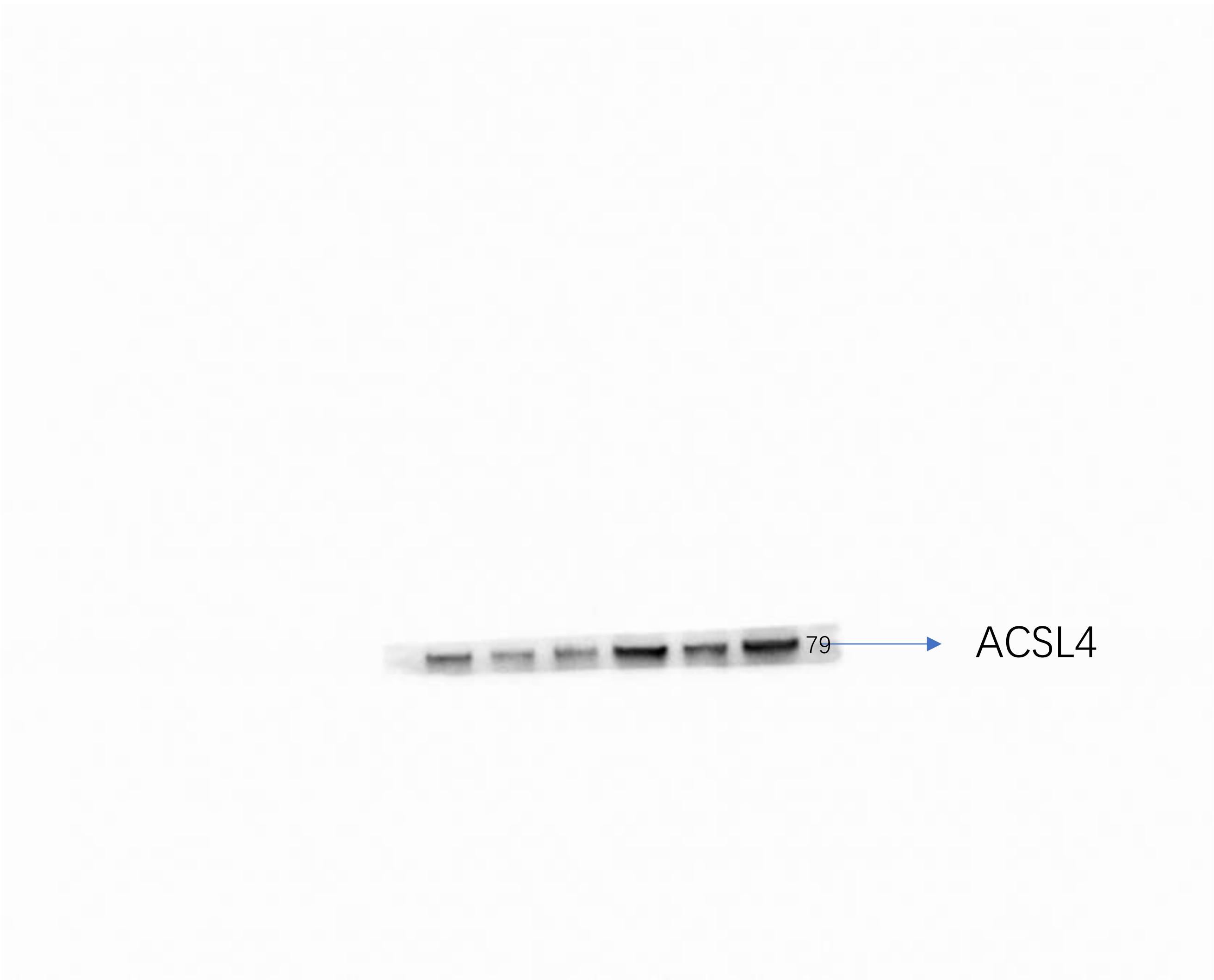

Result-4（4F)

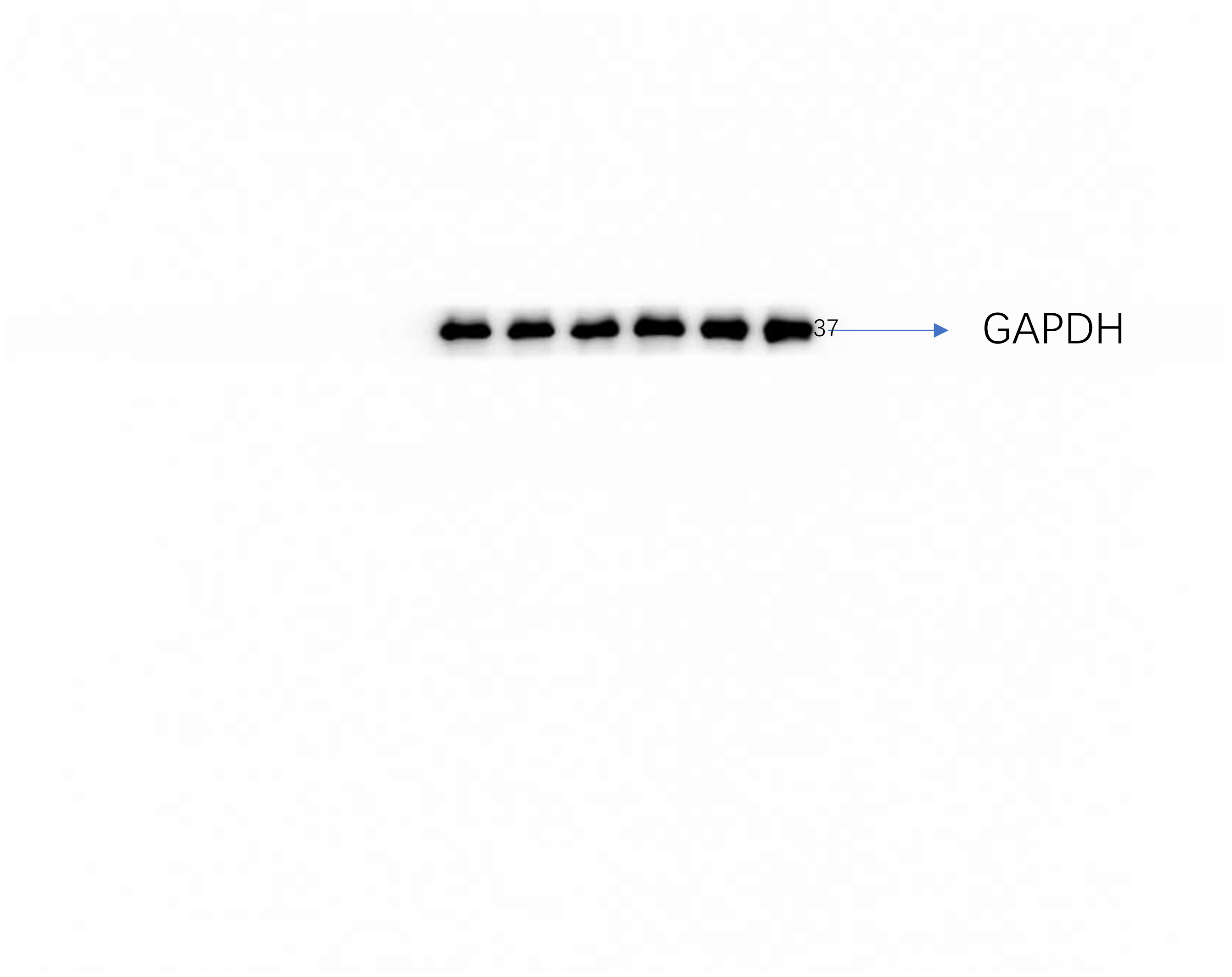

Result-4（4F）

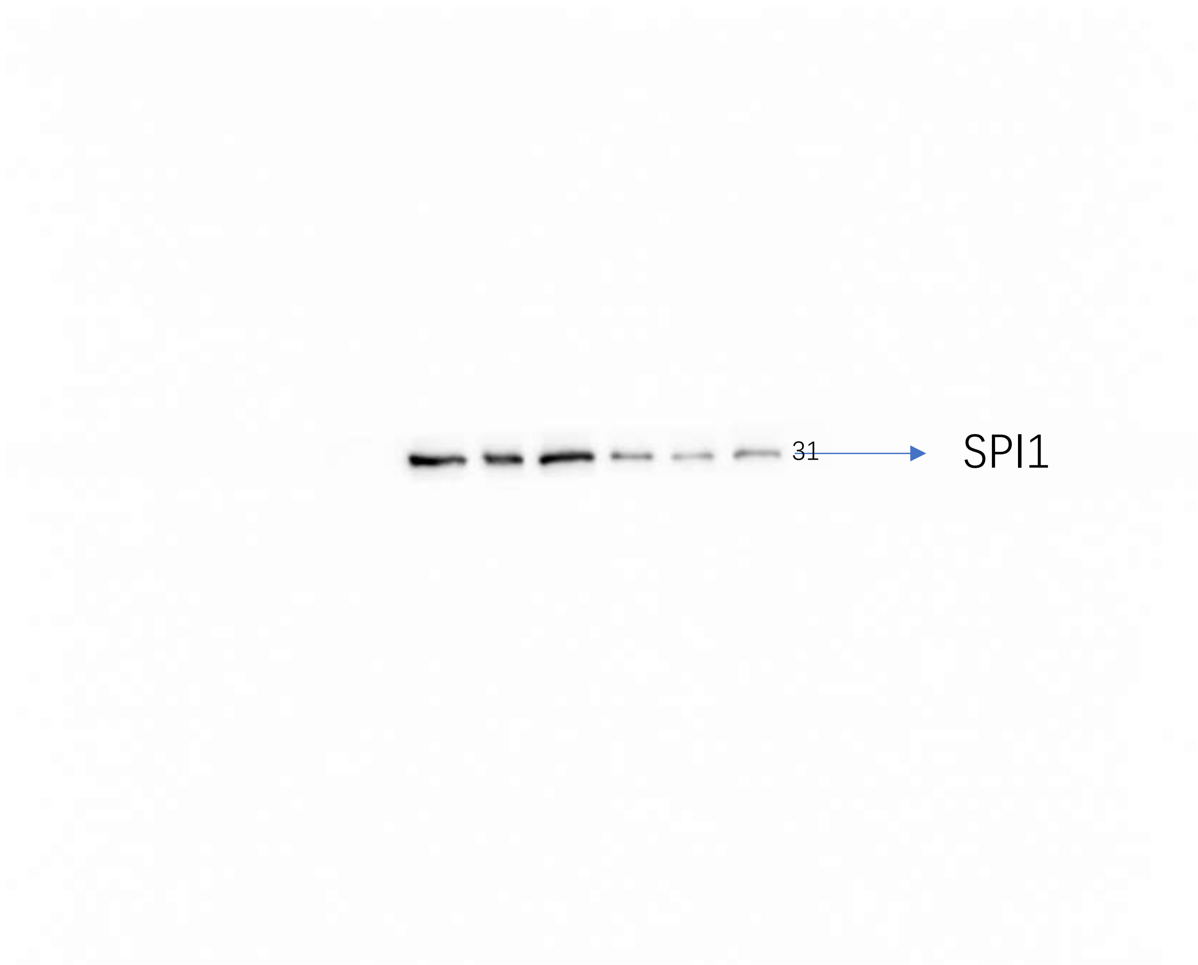

Result-5（5B）

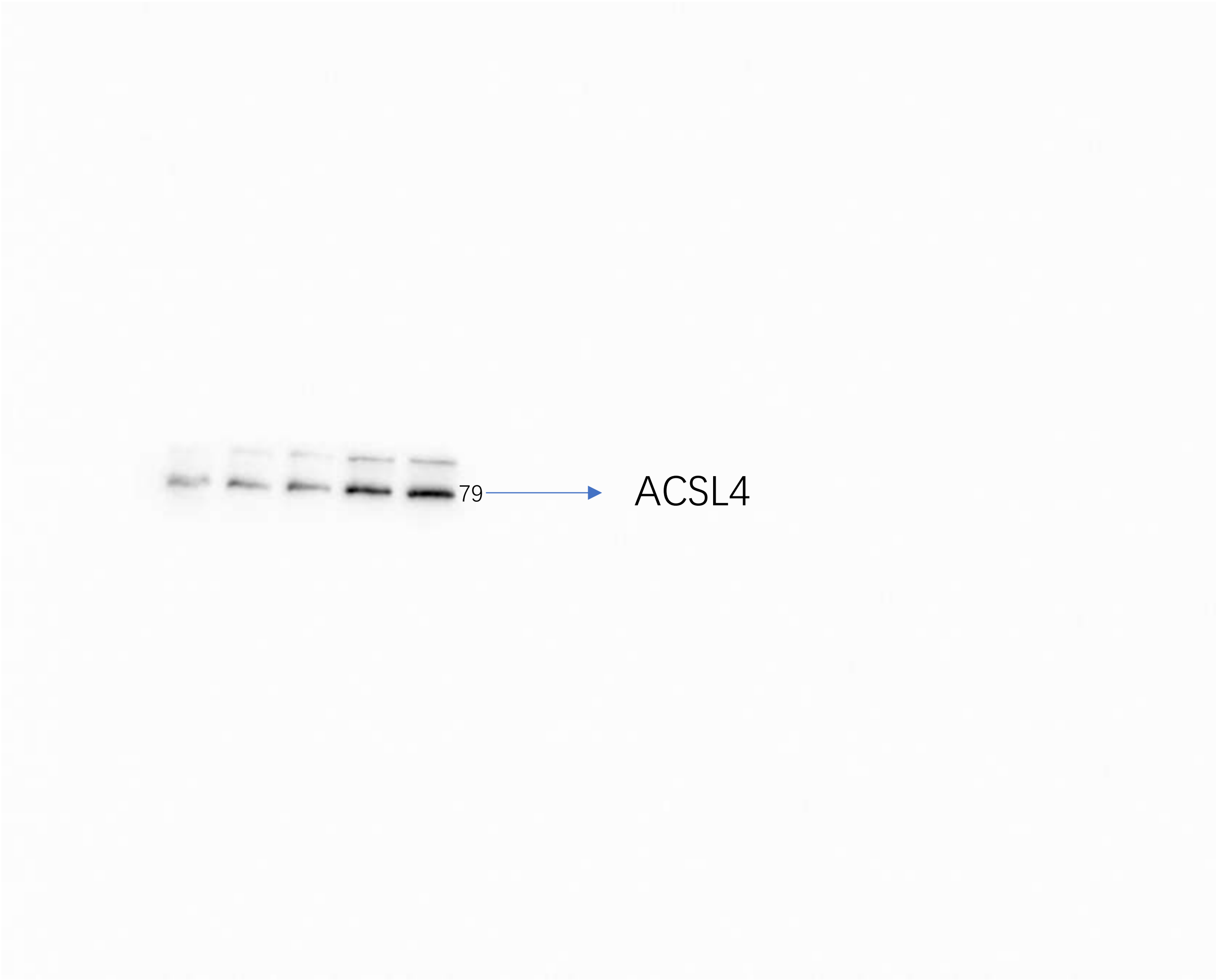

Result-5（5B）

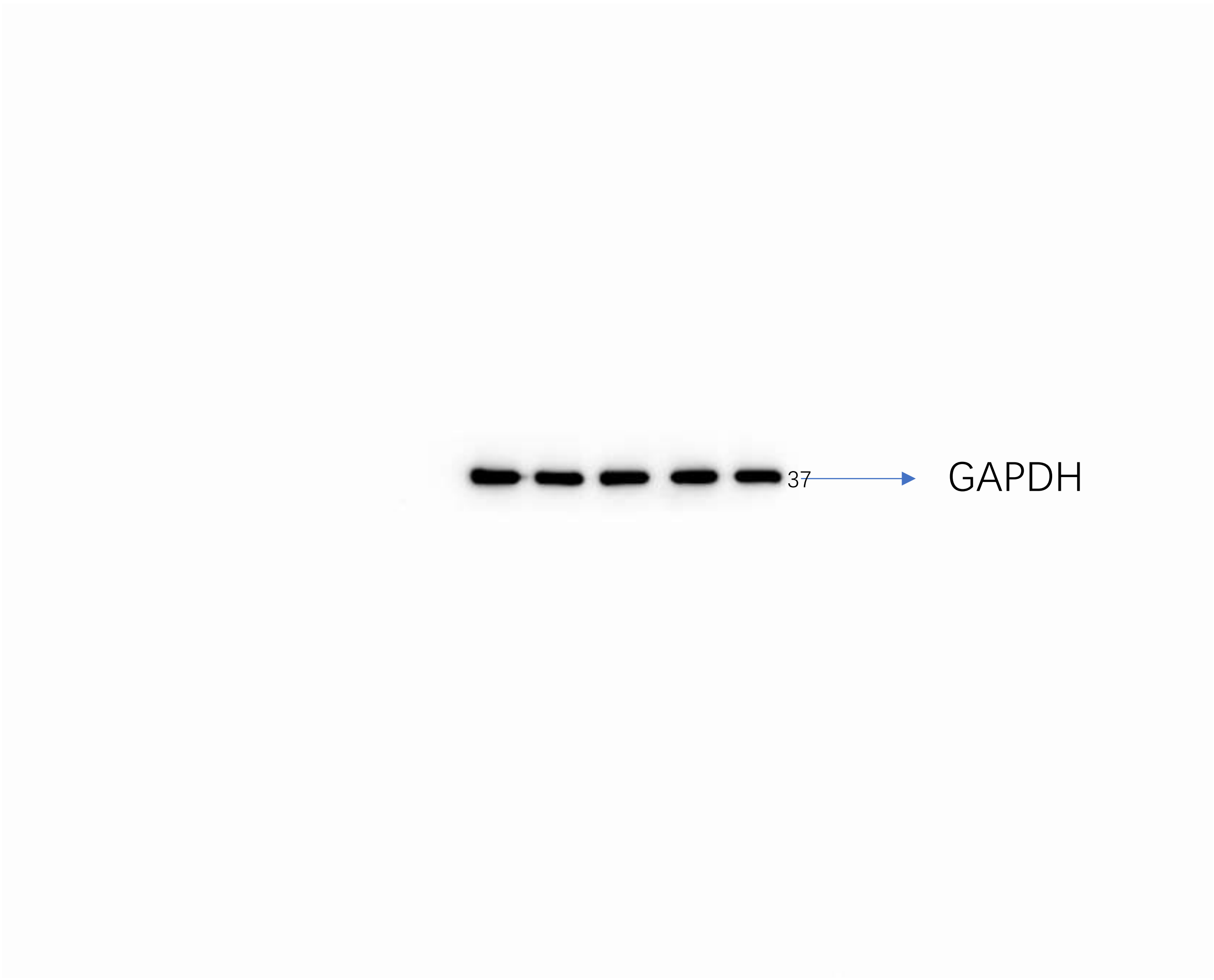

Result-5（5B）

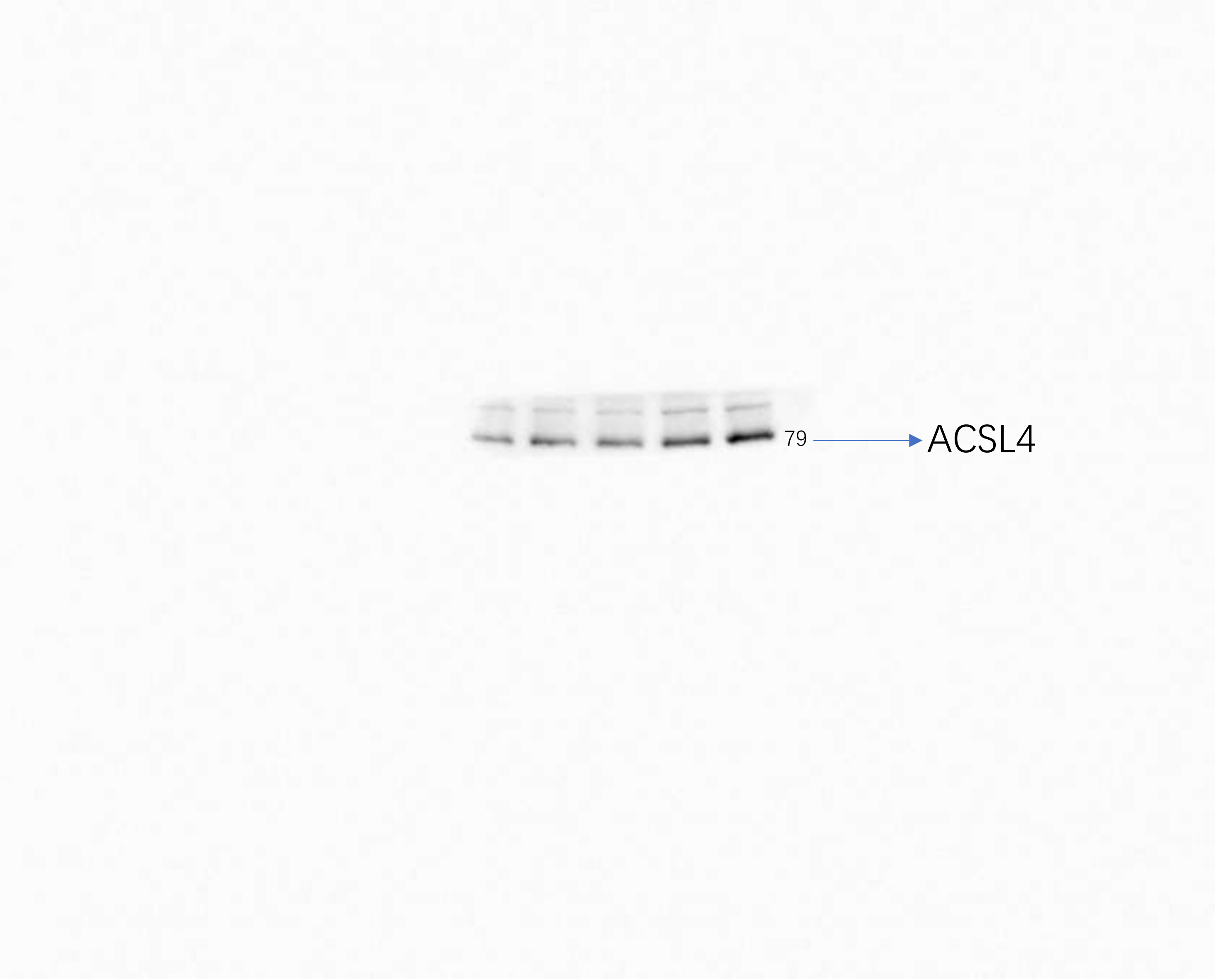

Result-5（5B)

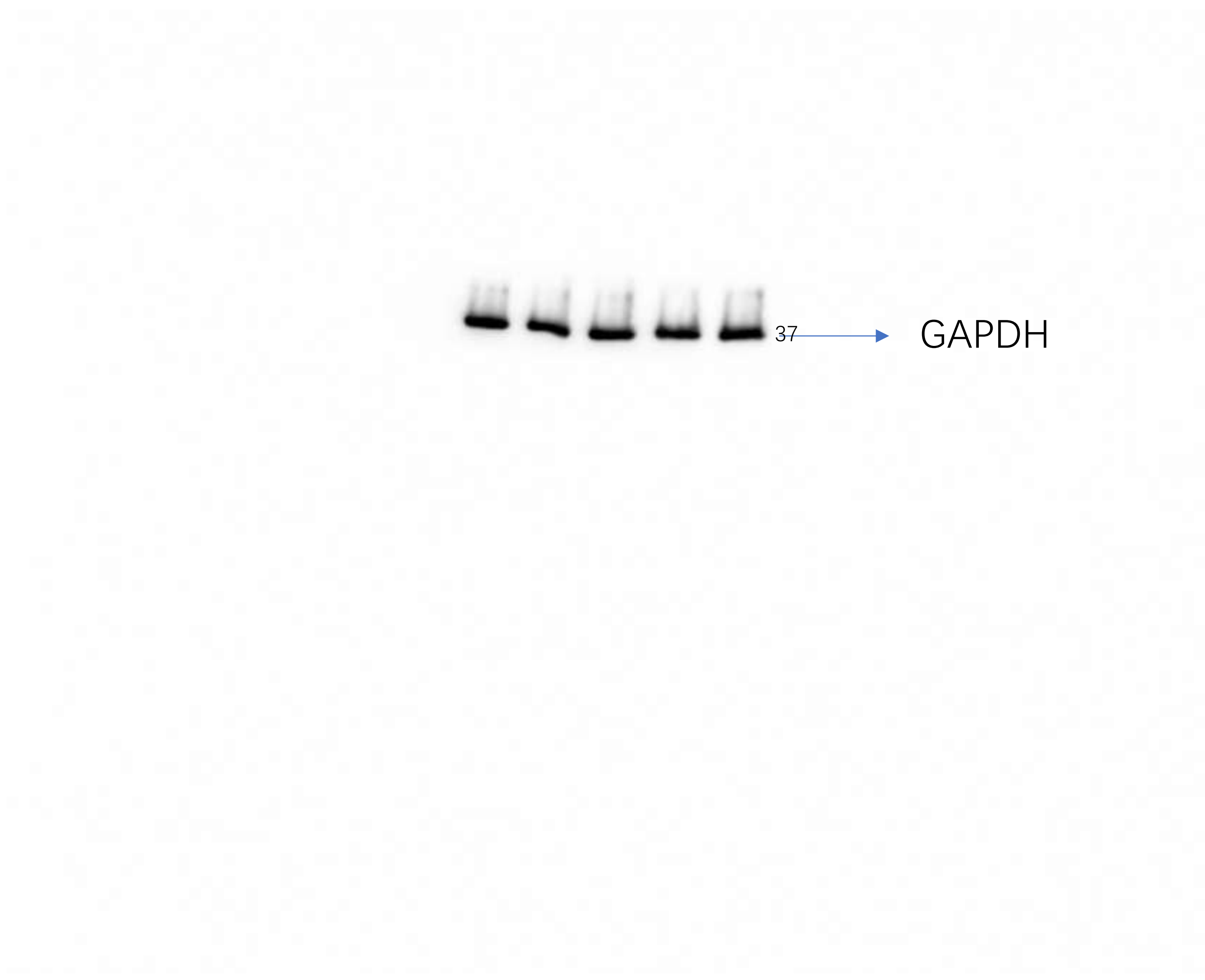

Result-5（5E）

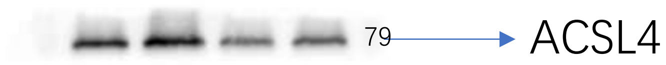

Result-5（5E）

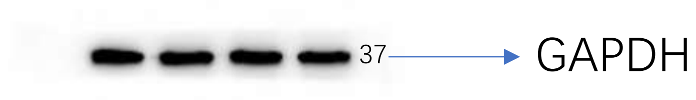

Result-5（5E)

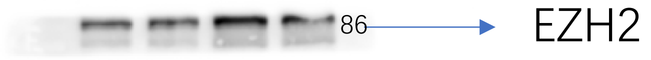

Result-5（5E）

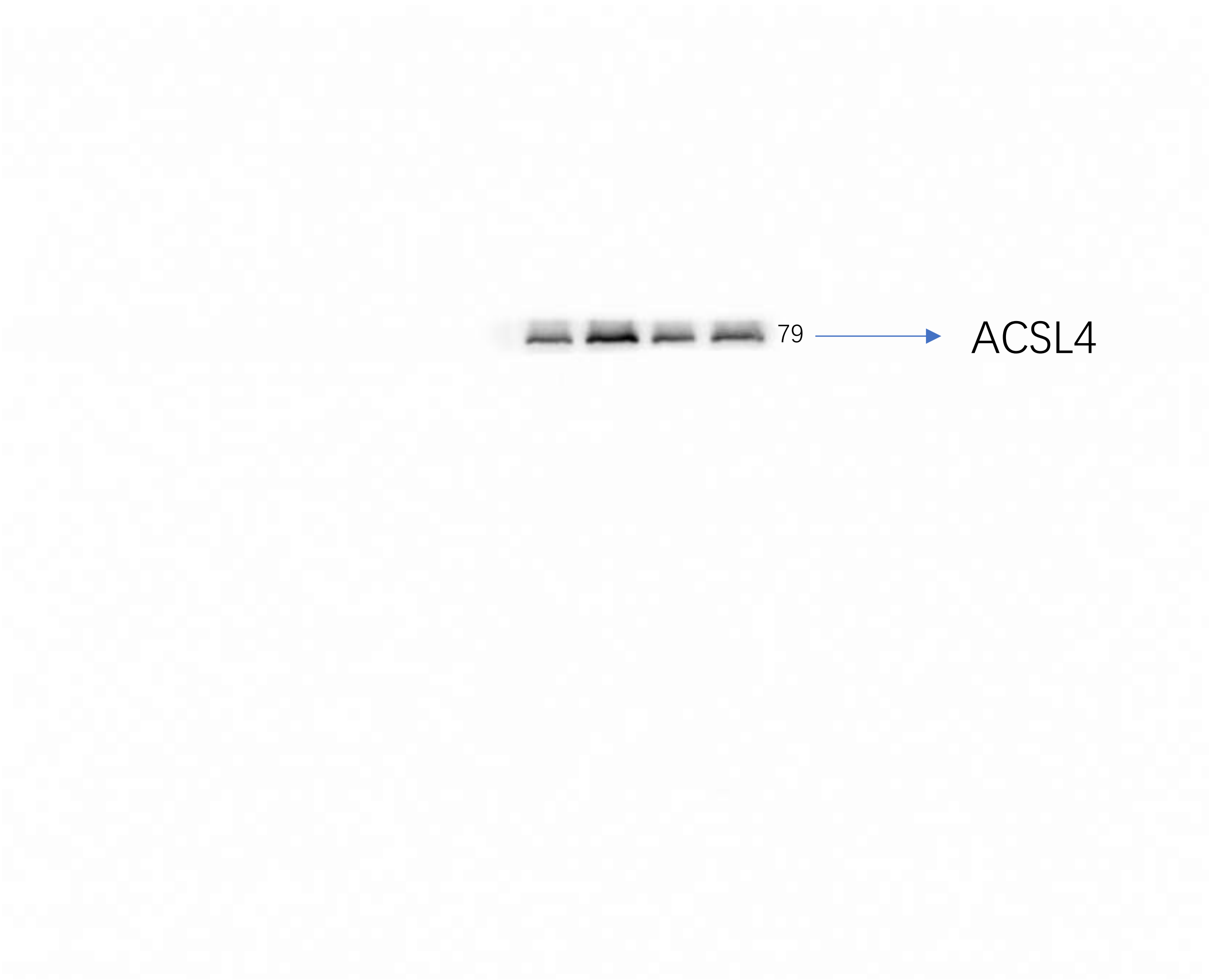

Result-5（5E）

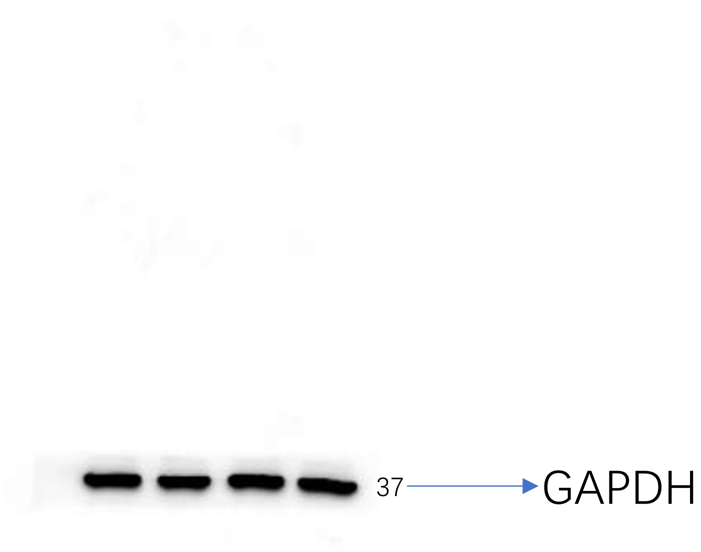

Result-5（5E)

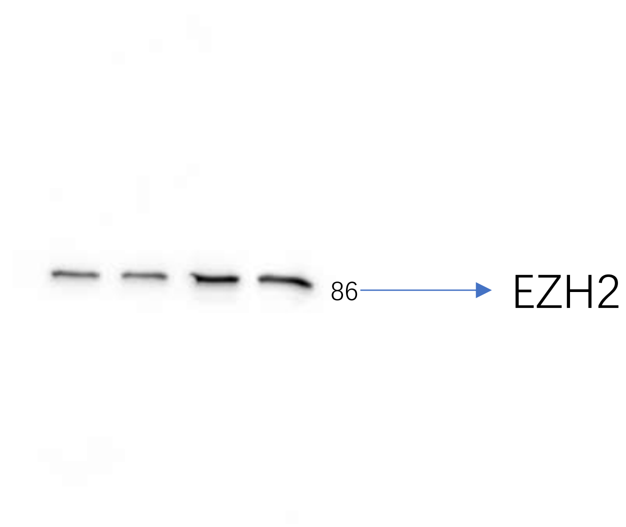

Result-5（5）

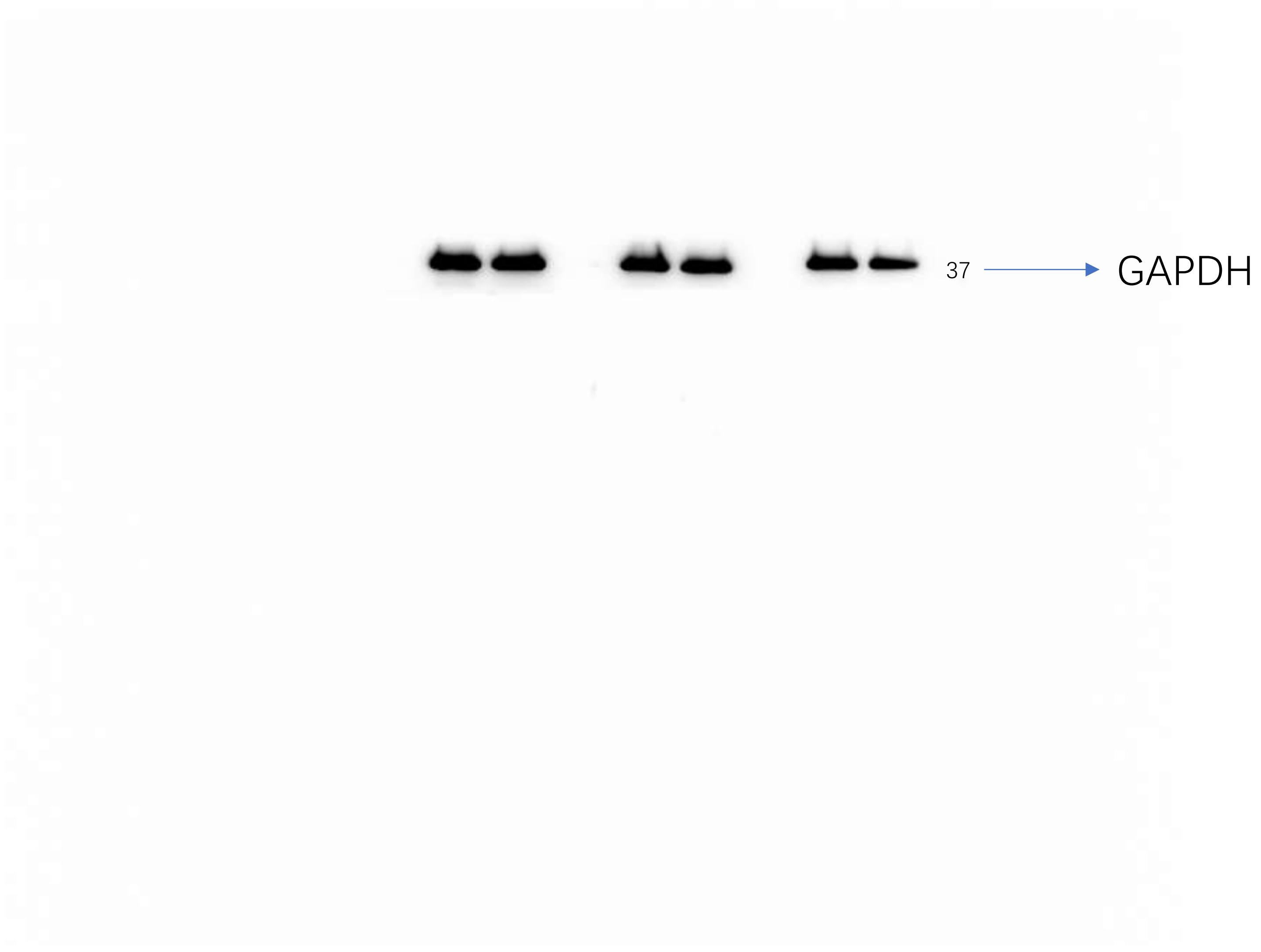

Result-5（5）

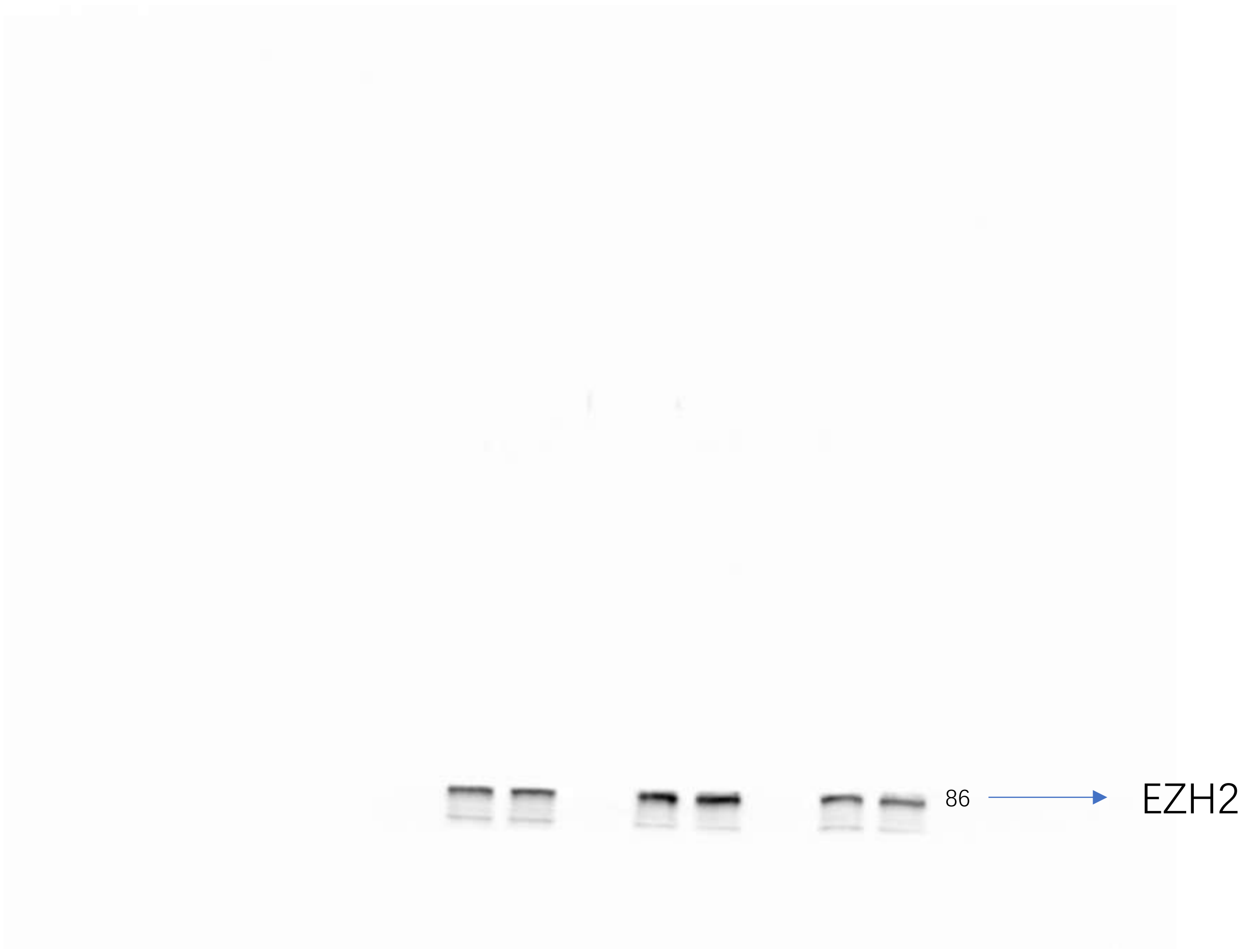

Result-5（5）

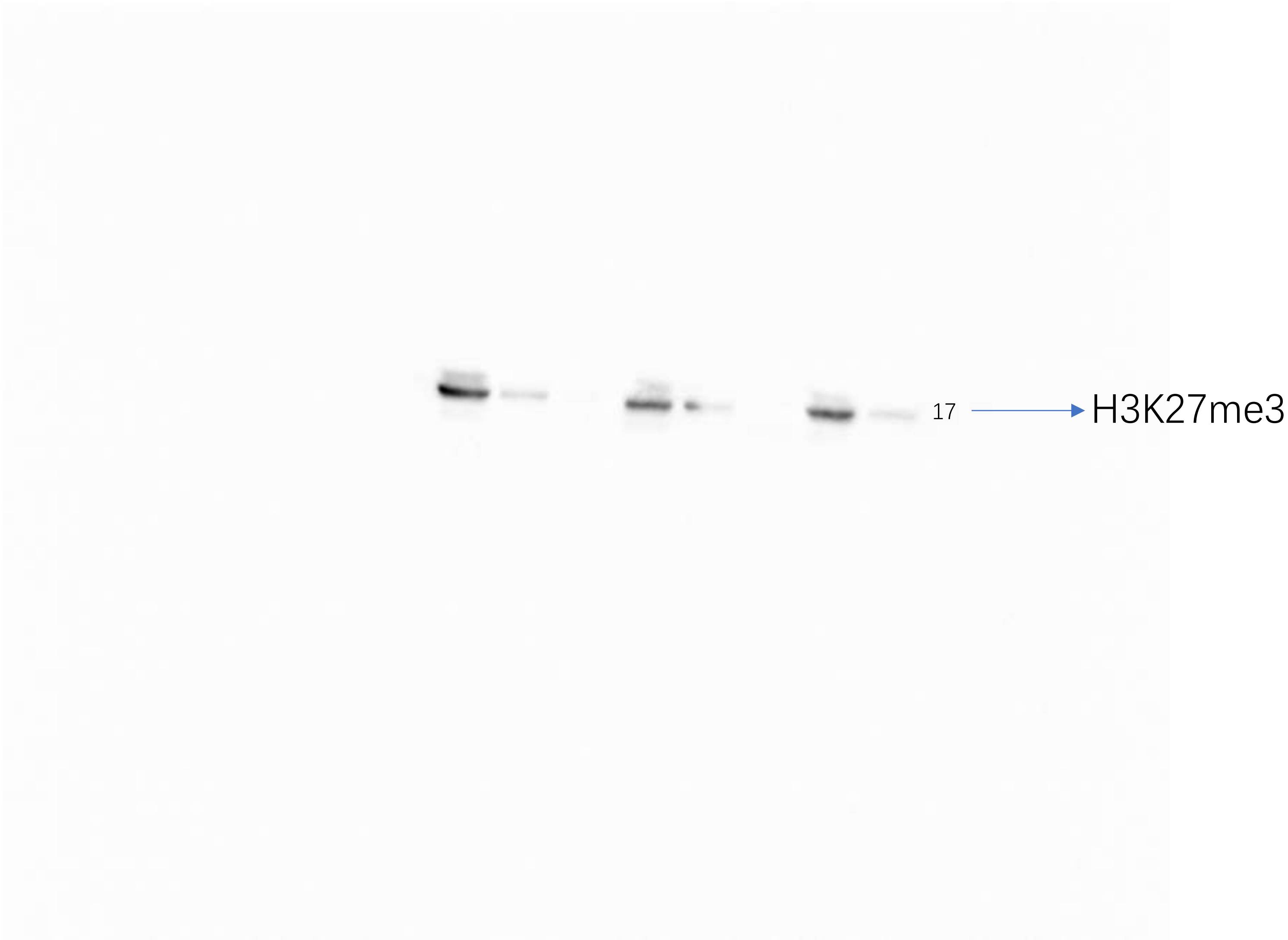

Result-5（5A）

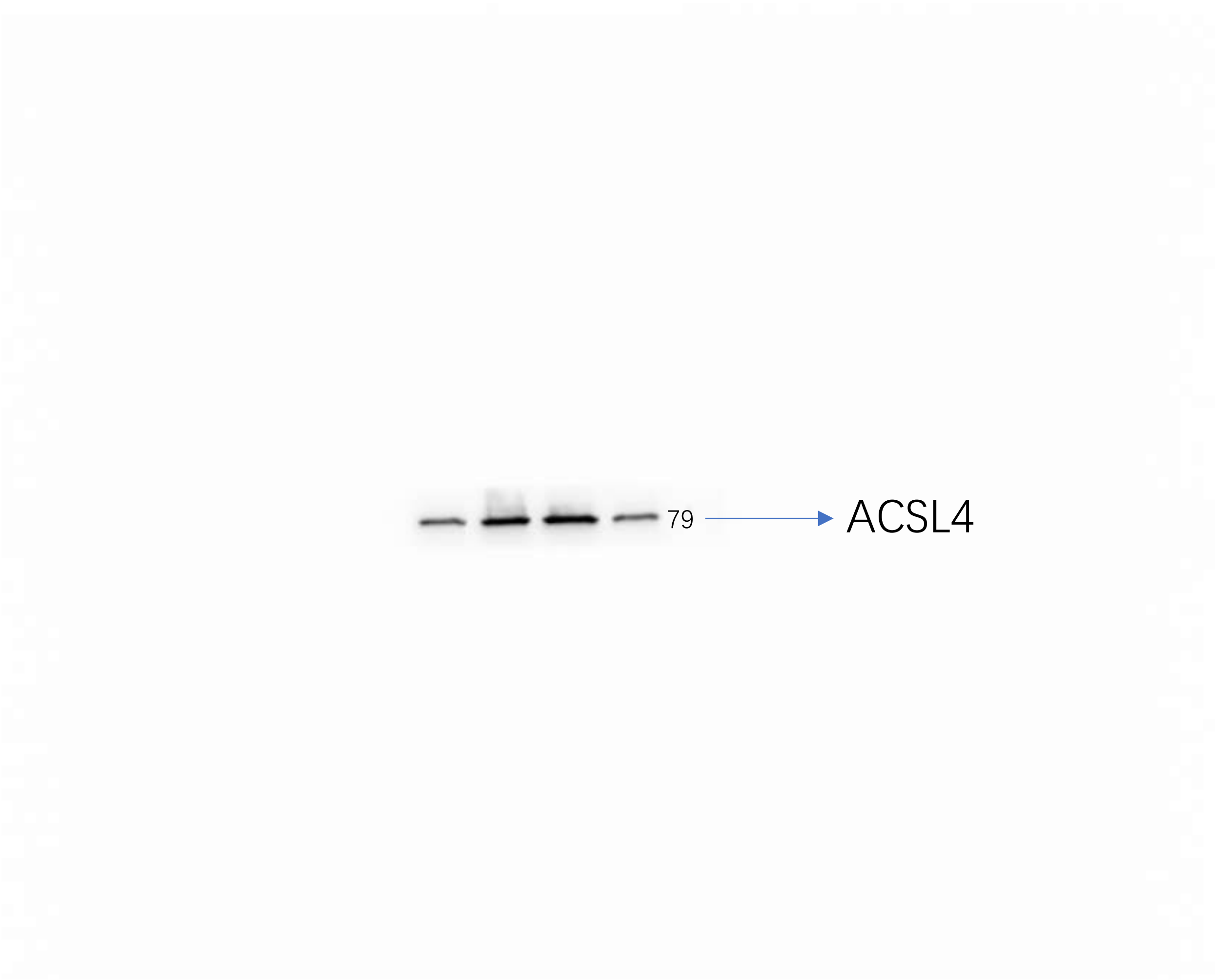

Result-5（5A）

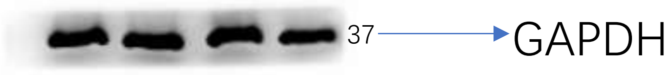

Result-5（5A）

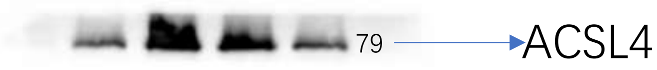

Result-5（5A)

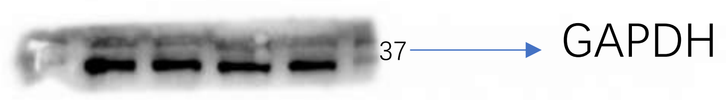

Result-5（5I）

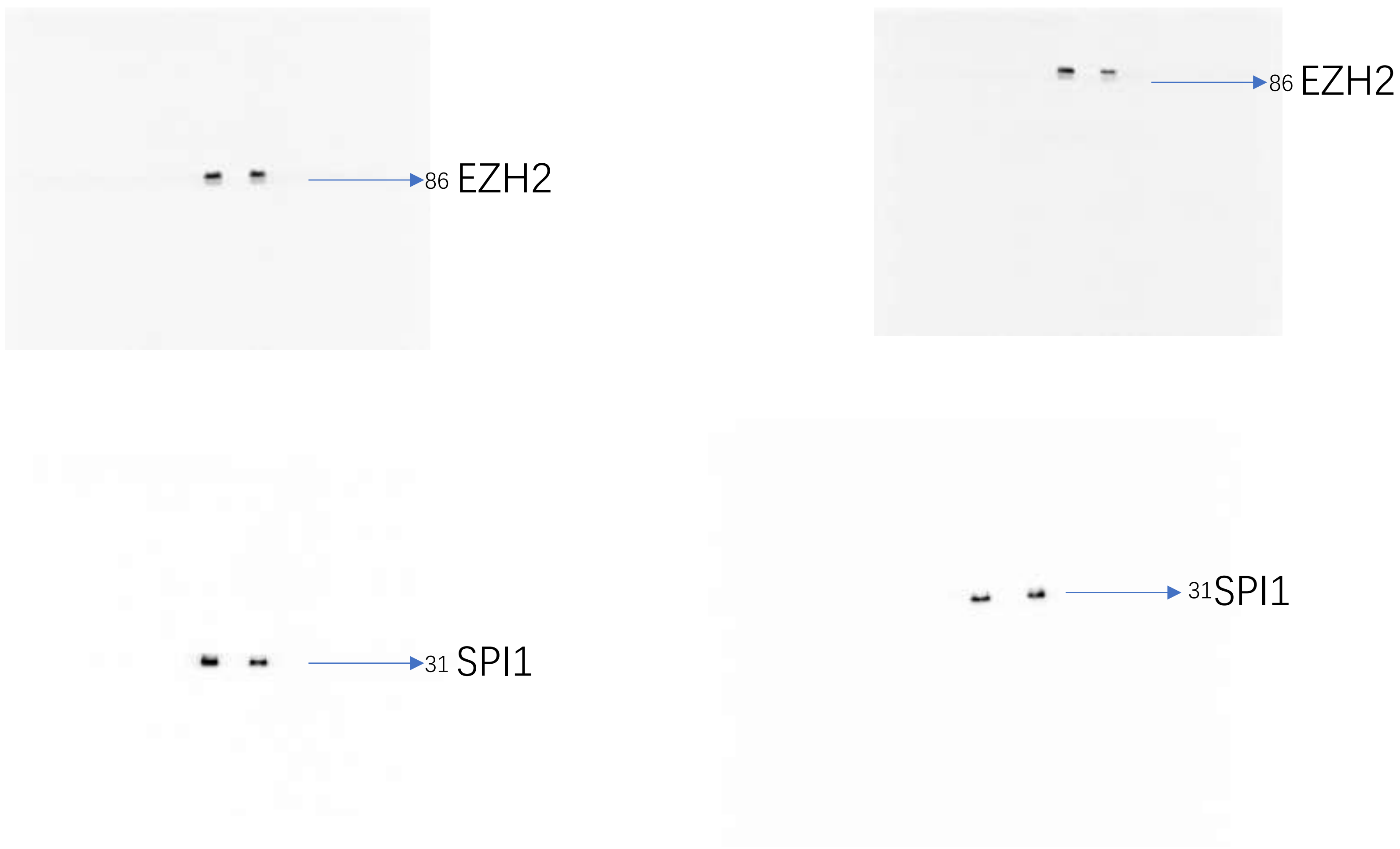

Result-5（5I）

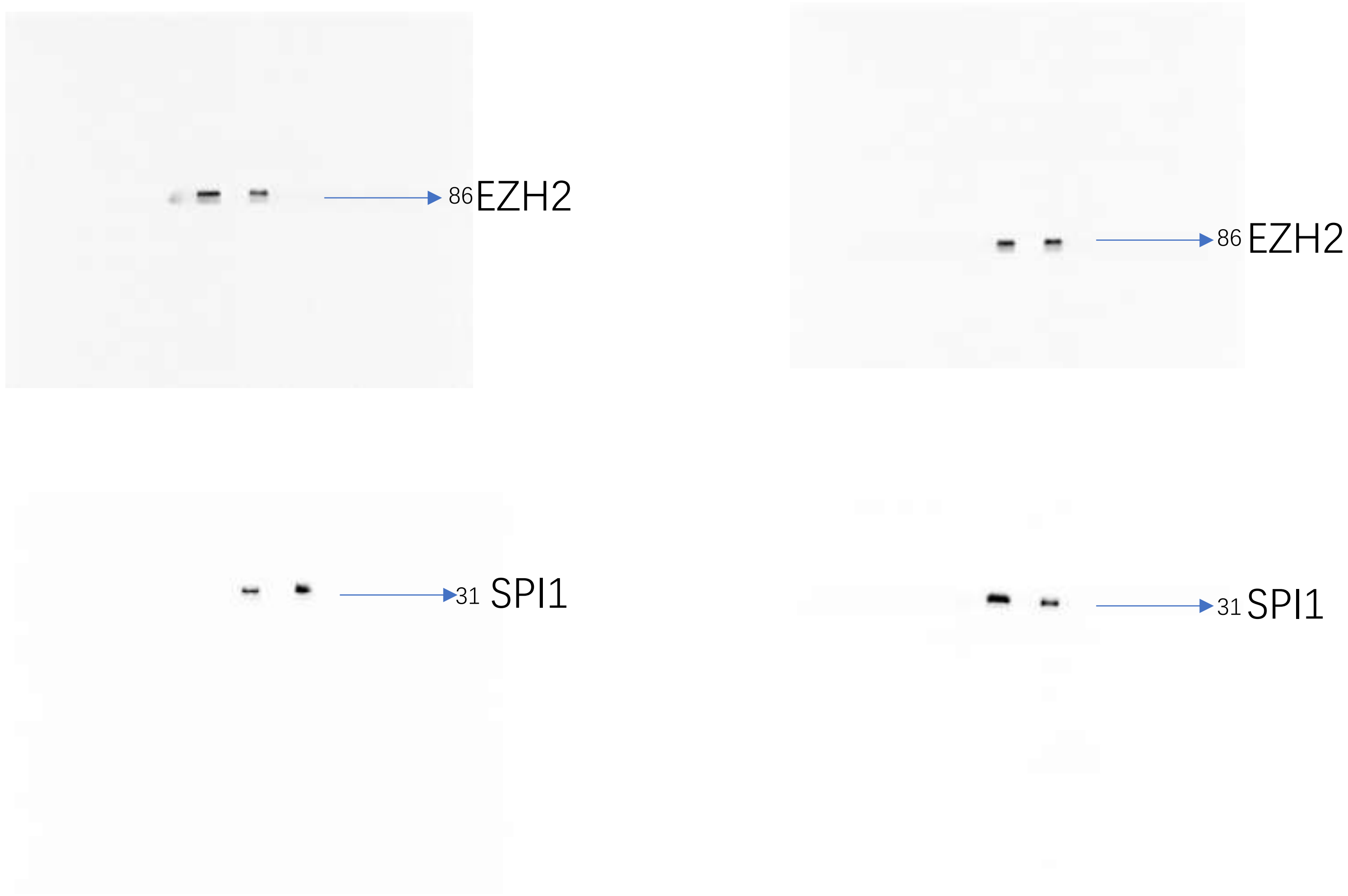

Result-7（7H）

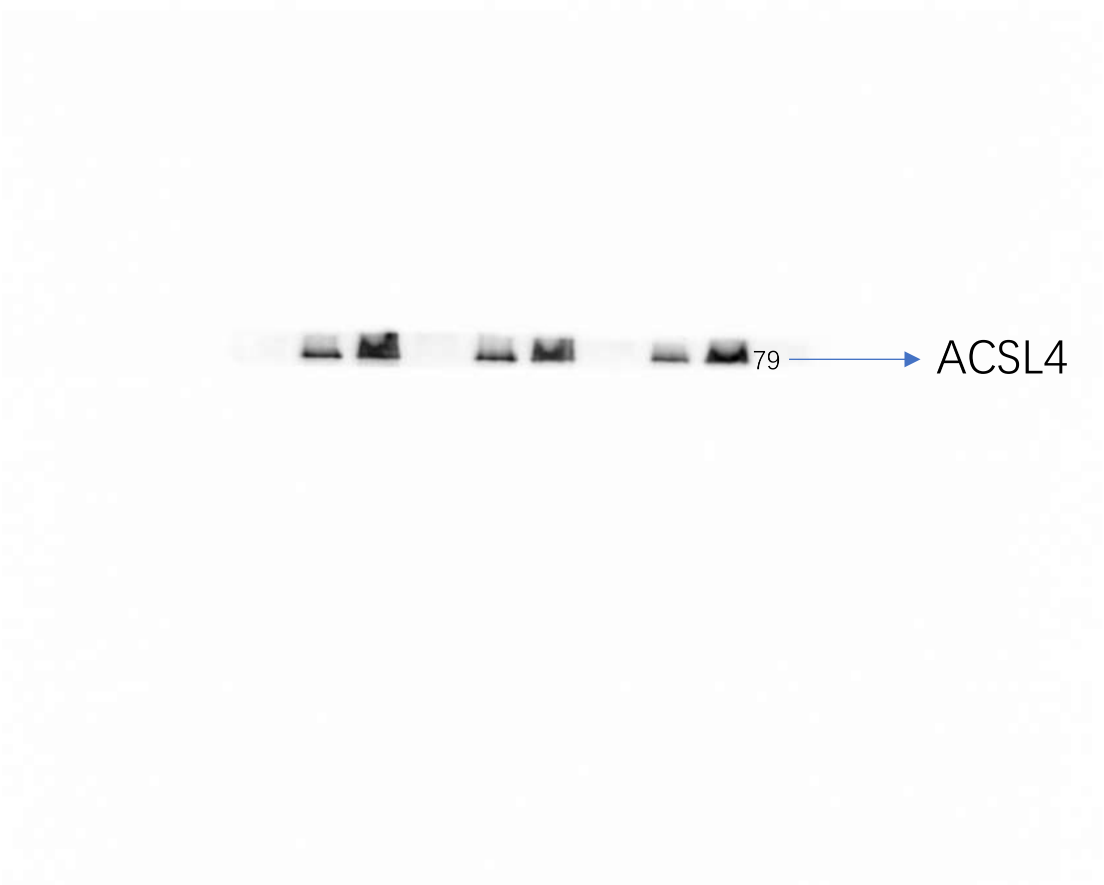

Result-7（7H）

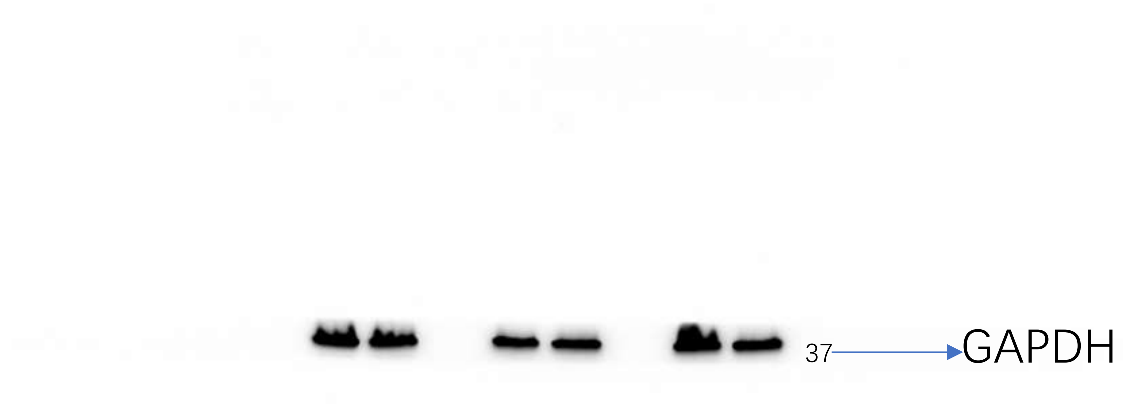

Result-8（8F）

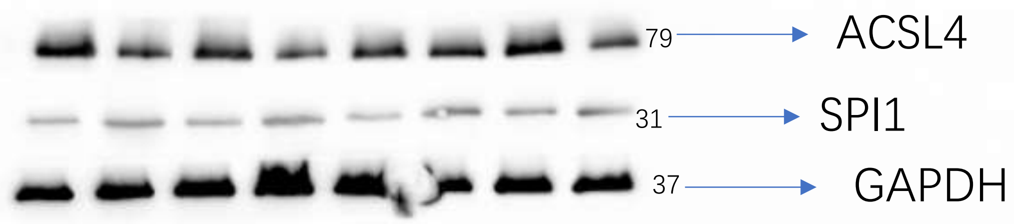

Result-8（8F）

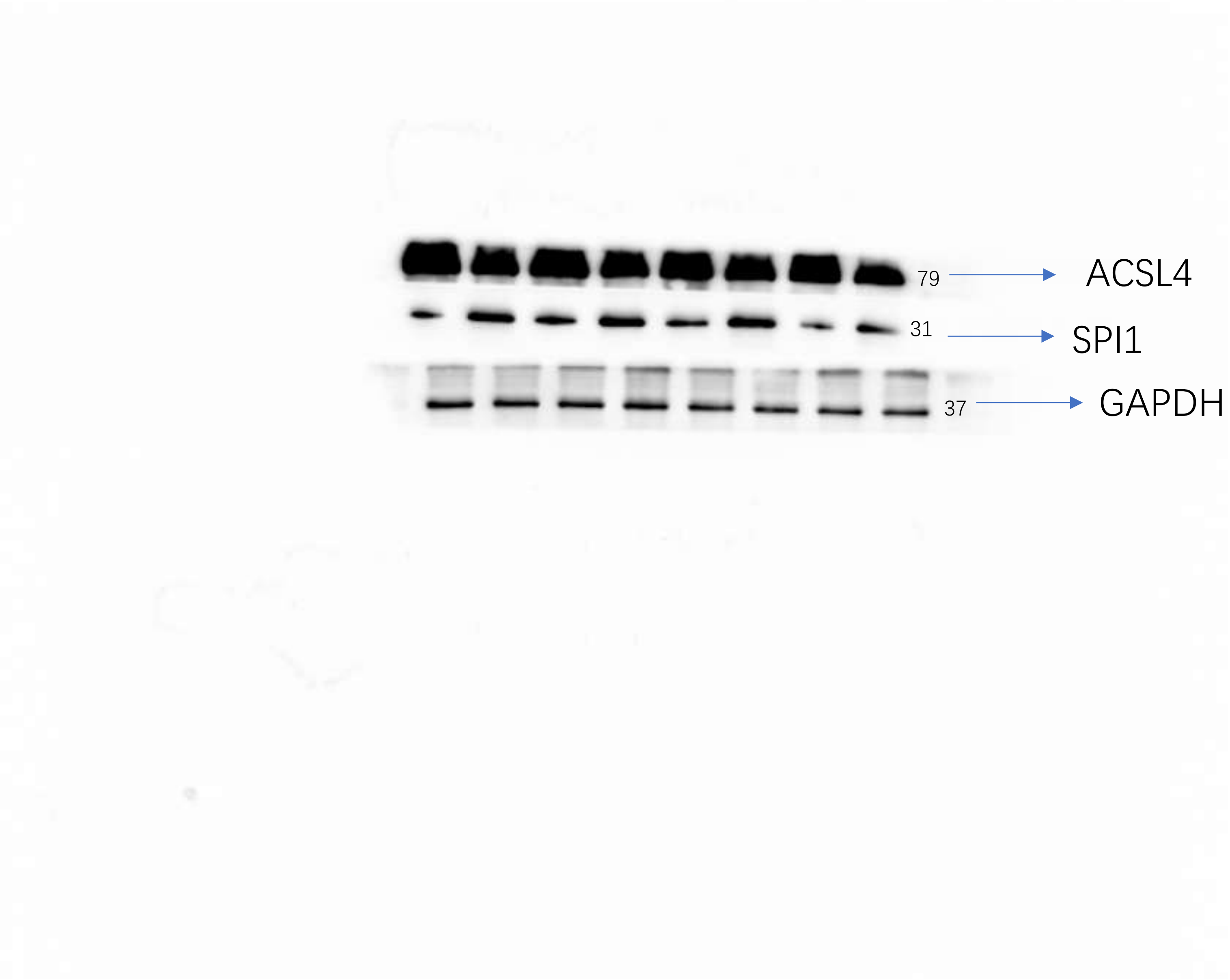

Result-8（8E）

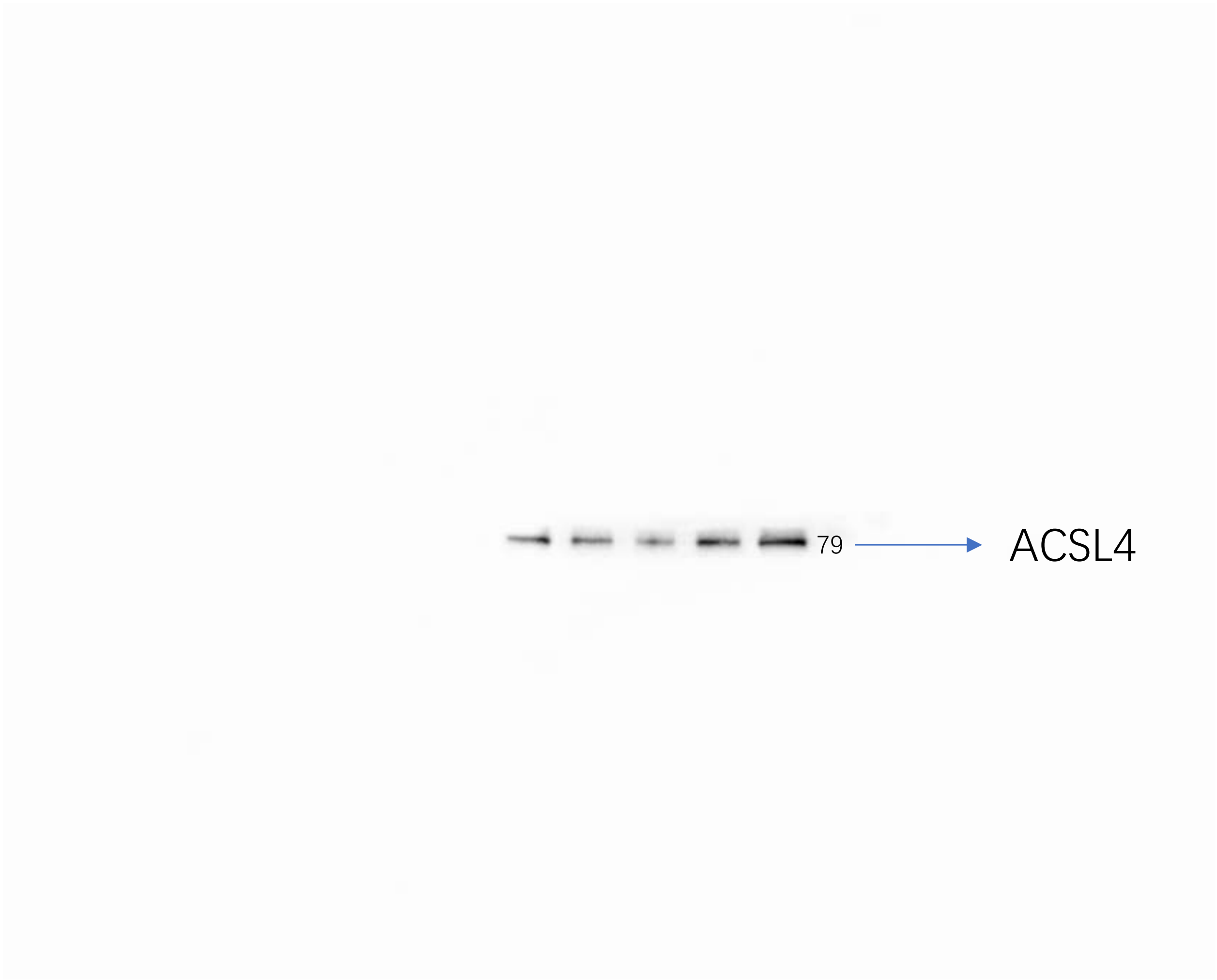

Result-8（8E）

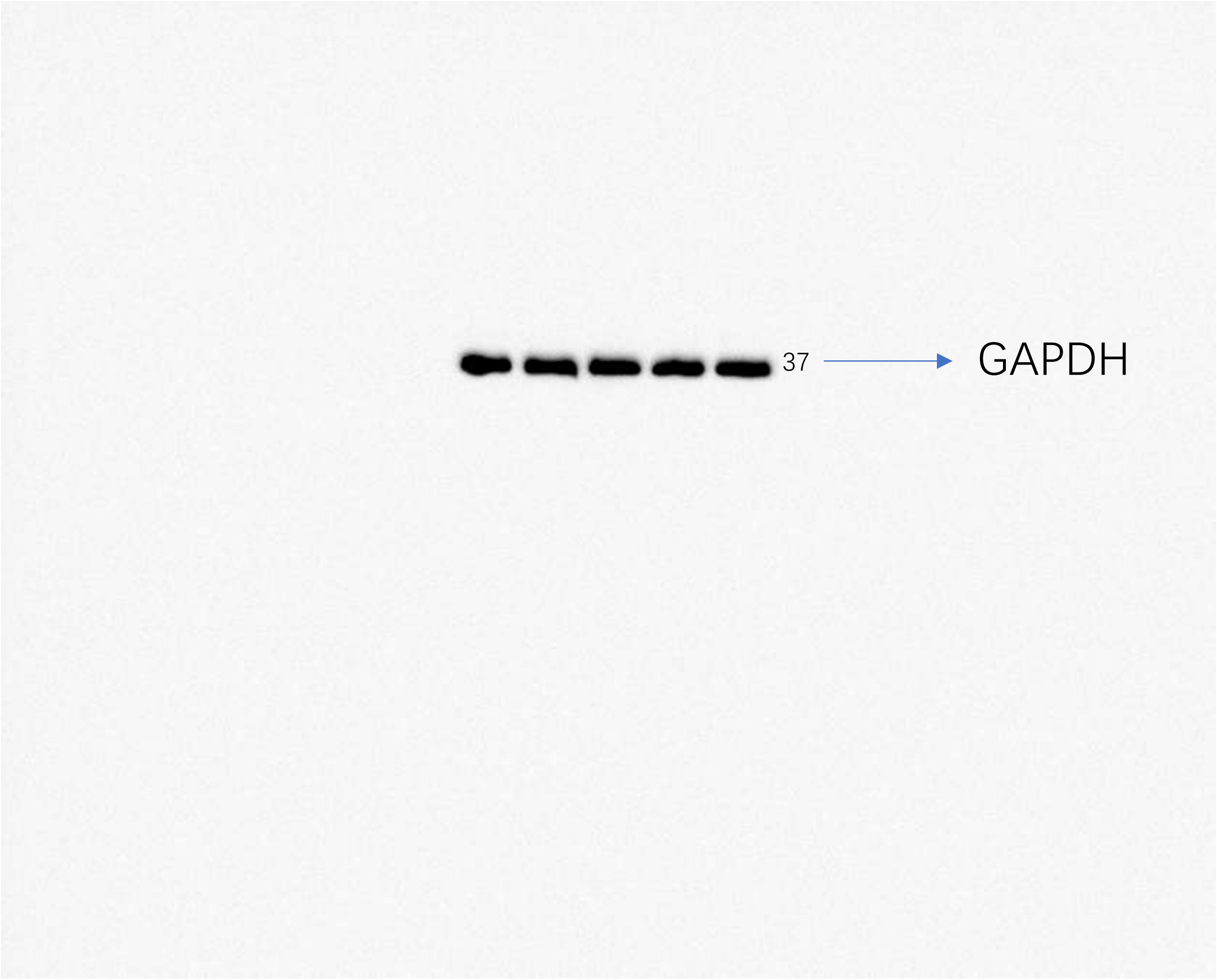

Result-8（8E)

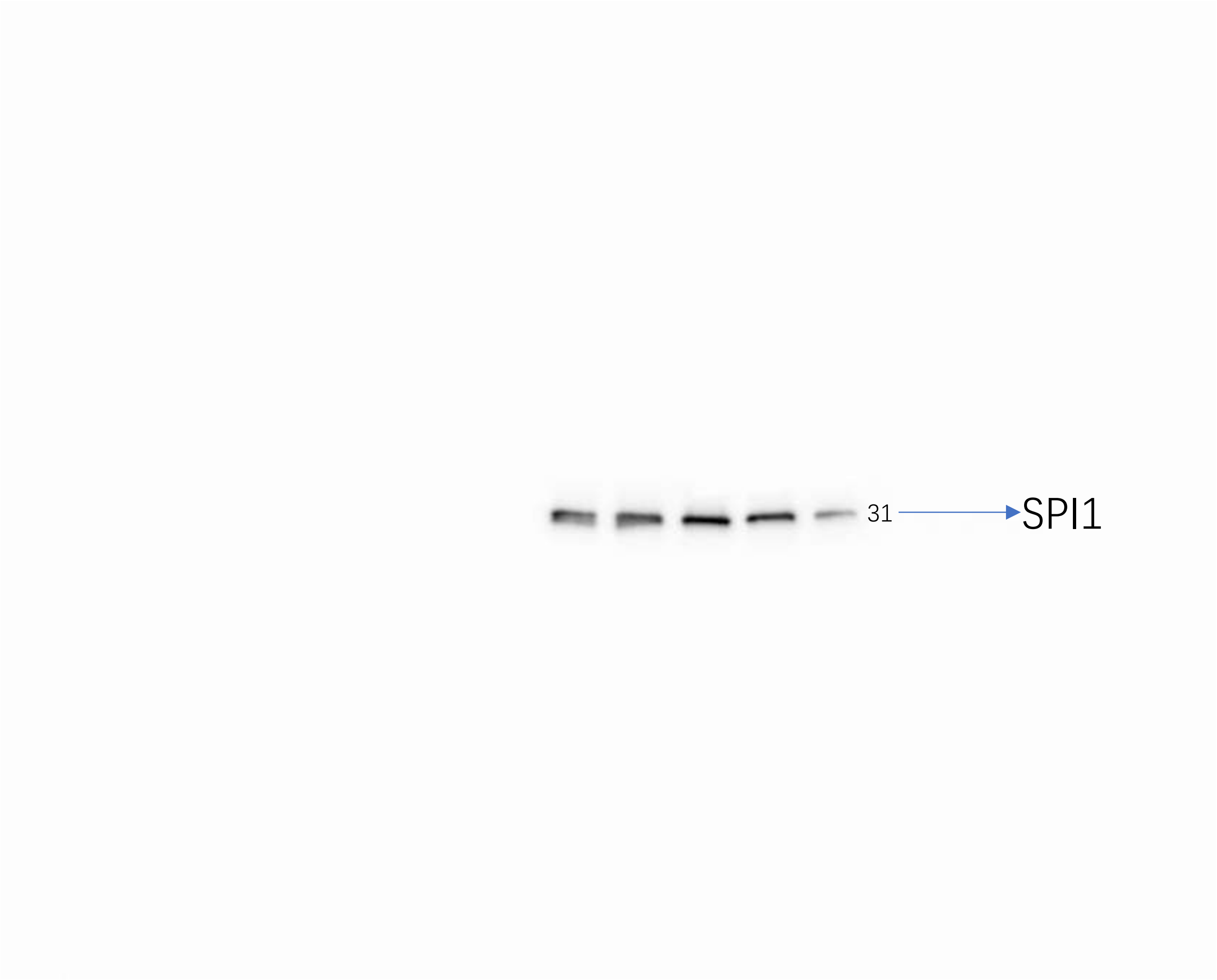

Supplement: Supplementary file 2 — Supplementary Information [file 42003_2025_8900_MOESM2_ESM.pdf]
