# Supplementary material for: Therapeutic targeting SPI1 in combination with erastin promotes ferroptosis in ccRCC
Source: Commun Biol. 2025 Dec 10;8:1772. doi: 10.1038/s42003-025-08900-4 (PMC12708875; doi:10.1038/s42003-025-08900-4)
Supplement: Supplementary file 3 — Description of Additional Supplementary Materials [file 42003_2025_8900_MOESM3_ESM.pdf]

Description of Additional Supplementary Files

File name: Supplementary Data 1

Description: The relevant gene siRNA sequences and primer sequences are provided as follows

File name: Supplementary Data 2

Description: The HumanTFDB website was used to predict the potential binding sites for EZH2 in the promoter of ACSL4

File name: Supplementary Data 3

Description: By analyzing the TCGA-KIRC database and intersecting it with the ENCODE database transcription factor dataset

File name: Supplementary Data 4

Description: The primer sequences used for the generation and confirmation of the mutant are listed
