# Supplementary material for: Therapeutic targeting SPI1 in combination with erastin promotes ferroptosis in ccRCC
Source: Commun Biol. 2025 Dec 10;8:1772. doi: 10.1038/s42003-025-08900-4 (PMC12708875; doi:10.1038/s42003-025-08900-4)
Supplement: Supplementary file 5 — Reporting summary [file 42003_2025_8900_MOESM5_ESM.pdf]

Corresponding author(s): Xiang Fei

Last updated by author(s): Jun 11, 2025

Reporting Summary

Nature Portfolio wishes to improve the reproducibility of the work that we publish. This form provides structure for consistency and transparency in reporting. For further information on Nature Portfolio policies, see our Editorial Policies and the Editorial Policy Checklist.

Statistics

For all statistical analyses, confirm that the following items are present in the figure legend, table legend, main text, or Methods section.

n/a	Confirmed	
☐	☒	The exact sample size (*n*) for each experimental group/condition, given as a discrete number and unit of measurement
☐	☒	A statement on whether measurements were taken from distinct samples or whether the same sample was measured repeatedly
☐	☒	The statistical test(s) used AND whether they are one- or two-sided<br>*Only common tests should be described solely by name; describe more complex techniques in the Methods section.*
☒	☐	A description of all covariates tested
☒	☐	A description of any assumptions or corrections, such as tests of normality and adjustment for multiple comparisons
☐	☒	A full description of the statistical parameters including central tendency (e.g. means) or other basic estimates (e.g. regression coefficient) AND variation (e.g. standard deviation) or associated estimates of uncertainty (e.g. confidence intervals)
☐	☒	For null hypothesis testing, the test statistic (e.g. *F*, *t*, *r*) with confidence intervals, effect sizes, degrees of freedom and *P* value noted<br>*Give P values as exact values whenever suitable.*
☒	☐	For Bayesian analysis, information on the choice of priors and Markov chain Monte Carlo settings
☒	☐	For hierarchical and complex designs, identification of the appropriate level for tests and full reporting of outcomes
☒	☐	Estimates of effect sizes (e.g. Cohen's *d*, Pearson's *r*), indicating how they were calculated

Our web collection on statistics for biologists contains articles on many of the points above.

Software and code

Policy information about availability of computer code

Data collection	We conducted experimental research using the NovoExpress-1.6.0 flow cytometer
Data analysis	GraphPad Prism (version: 7.0) was used for statistical data analysis. Experimental data are expressed as the mean±standard deviation (mean± SD) deviation. Data were analyzed by t -test (two groups' comparison) and Kruskal-Wallis test. (three or more groups' comparison). *: P<0.05, **: P<0.01, ***: P<0.001

For manuscripts utilizing custom algorithms or software that are central to the research but not yet described in published literature, software must be made available to editors and reviewers. We strongly encourage code deposition in a community repository (e.g. GitHub). See the Nature Portfolio guidelines for submitting code & software for further information.

Data

Policy information about availability of data

All manuscripts must include a data availability statement. This statement should provide the following information, where applicable:

- Accession codes, unique identifiers, or web links for publicly available datasets
- A description of any restrictions on data availability
- For clinical datasets or third party data, please ensure that the statement adheres to our policy

Provide your data availability statement here.

Research involving human participants, their data, or biological material

Policy information about studies with human participants or human data. See also policy information about sex, gender (identity/presentation), and sexual orientation and race, ethnicity and racism.

Reporting on sex and gender	This study does not involve research related to human gender.
Reporting on race, ethnicity, or other socially relevant groupings	This study does not involve research related to human variables.
Population characteristics	This study does not involve research related to humans.
Recruitment	No relevant personnel were recruited for this experiment.
Ethics oversight	This experiment utilized 30 randomly selected samples of clear cell renal cell carcinoma tissues for PCR and IHC analysis, with the approval of the Ethics Committee of Shengjing Hospital.

Note that full information on the approval of the study protocol must also be provided in the manuscript.

Field-specific reporting

Please select the one below that is the best fit for your research. If you are not sure, read the appropriate sections before making your selection.

☒ Life sciences ☐ Behavioural & social sciences ☐ Ecological, evolutionary & environmental sciences

For a reference copy of the document with all sections, see nature.com/documents/nr-reporting-summary-flat.pdf

Life sciences study design

All studies must disclose on these points even when the disclosure is negative.

Sample size	The sample size for each experiment is indicated in the figures and figure legends, and the number of samples was not predetermined by statistical calculations. The experimental samples and groupings were established based on previous laboratory experience.
Data exclusions	No samples or animals were excluded during the analysis.
Replication	The related experiments encompass numerous independent repetitive steps, with each experiment being replicated at least three times to ensure the reliability of the results.
Randomization	Four-week-old male mice were selected as recipients for xenograft transplantation and were randomly assigned to various experimental groups. Since the cell experiments already had clear group divisions (e.g., wild type vs. knockdown), randomization was not performed.
Blinding	For cell-based experiments, including Western blot and flow cytometry, the cell types were determined prior to the experiment, meaning that the cell types were prepared at the outset and at the beginning of the experiment. During the experiment and result evaluation, the researchers were not blinded. The experimental and control groups were clearly defined, thus blinding was not necessary.

Reporting for specific materials, systems and methods

We require information from authors about some types of materials, experimental systems and methods used in many studies. Here, indicate whether each material, system or method listed is relevant to your study. If you are not sure if a list item applies to your research, read the appropriate section before selecting a response.

Materials & experimental systems

n/a	Involved in the study	
☐	☒	Antibodies
☐	☒	Eukaryotic cell lines
☒	☐	Palaeontology and archaeology
☐	☒	Animals and other organisms
☒	☐	Clinical data
☒	☐	Dual use research of concern
☒	☐	Plants

Methods

n/a	Involved in the study	
☒	☐	ChIP-seq
☐	☒	Flow cytometry
☒	☐	MRI-based neuroimaging

Antibodies

Antibodies used	SPI1 (55100-1-AP, Proteintech), MCL1 (16225-1-AP, Proteintech), H3K27me3 (9733S, CST), Bcl-2 (68103-1-Ig, Proteintech), ACSL4 (22401-1-AP, Proteintech), EZH2 (21800-1-AP, Proteintech)?β-ACTIN (66009-1-Ig , Proteintech) and GAPDH?60004-1-Ig, Proteintech?. The anti-SPI1 antibody (55100-1-AP, Proteintech) and anti-ACSL4 antibody (22401-1-AP, Proteintech) for IHC were purchased from Proteintech. The Co-IP experimental antibodies were anti-SPI1 antibody (2266, CST) and EZH2 antibody (39076, mAb). The anti-H3K27me3 antibody (9733S), the anti-SPI1 antibody (2266) and EZH2 antibody (5246S) for ChIP-PCR were purchased from CST. Erastin (HY-15763)
Validation	All antibodies were proportioned according to the instructions, and the effects of the antibodies were validated accordingly.

Eukaryotic cell lines

Policy information about cell lines and Sex and Gender in Research

Cell line source(s)	Human normal renal cell lines HK-2 and renal cancer cell lines (786-O, ACHN, Caki-1, A498) were procured from the Cell Resources Center at the Shanghai Academy of Life Sciences, Chinese Academy of Sciences. The renal cell lines were maintained in the appropriate culture medium supplemented with 10% heat-inactivated fetal bovine serum (Thermo Fisher Scientific) and 1% streptomycin/penicillin (Keygen, Nanjing, China) at 37 °C and 5% CO2.
Authentication	Cell lines have not been identified.
Mycoplasma contamination	No mycoplasma contamination was detected in any cell line.
Commonly misidentified lines (See ICLAC register)	No ICLAC cell lines were used in this study.

Animals and other research organisms

Policy information about studies involving animals; ARRIVE guidelines recommended for reporting animal research, and Sex and Gender in Research

Laboratory animals	According to the experimental needs, we randomly divided 4-week-old male nude mice into four groups and raised them for a week to adapt to the environment and eliminate interference.
Wild animals	No wild animals were involved in this experiment.
Reporting on sex	According to the experimental needs, 4-week-old male nude mice were selected in this study.
Field-collected samples	(1) Temperature. The room temperature suitable for naked mice is 26~28°C (78~820F)<br><br>(2) Humidity. The relative temperature should be kept at 40~60%.<br>(3) Ventilation. It is generally believed that ventilation is required 10~15 times per hour.<br>(4) Lighting. A light and dark cycle of 10 hours of light and 14 hours of no light should be maintained every day.<br>In this animal experiment, nude mice were finally euthanized
Ethics oversight	This animal experiment meets the relevant ethical requirements and is approved by the Ethics Committee of Shengjing Hospital Affiliated to China Medical University.

Note that full information on the approval of the study protocol must also be provided in the manuscript.

Plants

Seed stocks	*Report on the source of all seed stocks or other plant material used. If applicable, state the seed stock centre and catalogue number. If plant specimens were collected from the field, describe the collection location, date and sampling procedures.*
Novel plant genotypes	*Describe the methods by which all novel plant genotypes were produced. This includes those generated by transgenic approaches, gene editing, chemical/radiation-based mutagenesis and hybridization. For transgenic lines, describe the transformation method, the number of independent lines analyzed and the generation upon which experiments were performed. For gene-edited lines, describe the editor used, the endogenous sequence targeted for editing, the targeting guide RNA sequence (if applicable) and how the editor was applied.*
Authentication	*Describe any authentication procedures for each seed stock used or novel genotype generated. Describe any experiments used to assess the effect of a mutation and, where applicable, how potential secondary effects (e.g. second site T-DNA insertions, mosiacism, off-target gene editing) were examined.*

Flow Cytometry

Plots

Confirm that:

☐ The axis labels state the marker and fluorochrome used (e.g. CD4-FITC).

☐ The axis scales are clearly visible. Include numbers along axes only for bottom left plot of group (a 'group' is an analysis of identical markers).

☐ All plots are contour plots with outliers or pseudocolor plots.

☐ A numerical value for number of cells or percentage (with statistics) is provided.

Methodology

Sample preparation	treated renal cancer cells or nude mouse tumors were added to the dye under light-avoidance conditions for 30 min. After three subsequent cold PBS washes, C11-BODIPY green/red fluorescence (510 nm and 590 nm) was detected by flow cytometry.
Instrument	NovoExpress-1.6.0
Software	The instrument processes the data itself during the experiment
Cell population abundance	*Describe the abundance of the relevant cell populations within post-sort fractions, providing details on the purity of the samples and how it was determined.*
Gating strategy	*Describe the gating strategy used for all relevant experiments, specifying the preliminary FSC/SSC gates of the starting cell population, indicating where boundaries between "positive" and "negative" staining cell populations are defined.*

☐ Tick this box to confirm that a figure exemplifying the gating strategy is provided in the Supplementary Information.
